# Men’s perception of paternal parenthood and the promotion of child development

**DOI:** 10.1590/0034-7167-2023-0514

**Published:** 2024-07-29

**Authors:** Gustavo Selenko de Aquino, Sarah de Almeida Rocha Moura, Ailton de Lima, Samara Macedo Cordeiro, Jéssica Batistela Vicente, Verônica de Azevedo Mazza

**Affiliations:** I Complexo Hospital de Clínicas e Maternidade Victor Ferreira do Amaral - filiado a EBSERH, Unidade da Mulher. Curitiba, Paraná, Brazil; II Irmandade da Santa Casa de Misericórdia. Curitiba, Paraná, Brazil; III Prefeitura Municipal de Curitiba. Curitiba, Paraná, Brazil; IV Universidade Estadual de Campinas. Campinas, São Paulo, Brazil; V Universidade Federal do Paraná. Curitiba, Paraná, Brazil

**Keywords:** Parenting, Paternity, Child Care, Child Development, Infant, Responsabilidad Parental, Paternidad, Cuidado del Niño, Desarrollo Infantil, Lactante

## Abstract

**Objectives:**

to comprehend men’s perception of paternal parenthood while caring for infants to promote child development.

**Methods:**

this qualitative study adopts an exploratory approach and was conducted with undergraduate and graduate students, faculty, and staff who are fathers of infants up to 6 months old from a higher education institution, excluding fathers from mononuclear families. Data collection occurred through semi-structured interviews and was analyzed using thematic categorical analysis.

**Results:**

fifteen men participated in the study. From the analysis, two empirical categories emerged: “Perception of being a father: challenges and novelties” and “Promotion of child development: actions carried out by fathers”. Fathers revealed feeling unprepared, the need for emotional support, and recognized activities aimed at their children’s development.

**Final Considerations:**

the relevance of the paternal figure for child development is highlighted, as well as the need for public policies to encourage paternal parenthood.

## RESUMEN

**Objetivos:**

comprender la percepción de los hombres en relación con la paternidad ejercida en el cuidado de los lactantes para promover el desarrollo infantil.

**Métodos:**

estudio cualitativo, de enfoque exploratorio, realizado con estudiantes, profesores y personal administrativo que son padres de lactantes de hasta 6 meses de edad de una institución de educación superior, excluyendo a padres de familias mononucleares. La recolección de datos se realizó a través de entrevistas semiestructuradas y se analizó bajo el enfoque del análisis temático categorial.

**Resultados:**

participaron en la investigación 15 hombres. A partir del análisis, surgieron dos categorías empíricas: “Percepción sobre ser padre: desafíos y novedades” y “Promoción del desarrollo infantil: acciones realizadas por los padres”. Los padres revelaron sentirse poco preparados, la necesidad de apoyo emocional y reconocen las actividades promovidas para el desarrollo de sus hijos.

**Conclusiones:**

se destaca la importancia de la figura paterna para el desarrollo infantil y la necesidad de políticas públicas que fomenten la paternidad activa.

## INTRODUCTION

Child development is an active and unique process for each child, evidenced by ongoing changes in motor, cognitive, socioemotional, and language skills, with progressive gains in abilities, from the simplest to the most complex, in daily life functions, and in the exercise of their social role^(1)^. Early childhood (0 to 6 years of age)^(2)^ has been recognized as a strategic moment for human development. During this period, the child’s brain undergoes intense expansion and has high plasticity^(3)^. This is the period of greatest risk when the child is exposed to adverse situations affecting their development, but it is also the phase with the best outcomes from actions promoting motor, cognitive, language, and socioemotional skills^(4-5)^.

Child development is influenced by the environment and context in which the child is raised. Thus, the absence of the maternal or paternal figure can lead to negative consequences for the child’s overall development throughout life^(6-7)^. Among these consequences, delays and difficulties in speech, social interaction, logical reasoning, adherence to physical activities, difficulty in concentration, and emotional maturity are observed^(1)^.

Regarding the paternal figure, it is noted that men sustain and facilitate the relationship between mother and child, both materially and emotionally. Materially, men provide financial and practical aspects, such as ensuring basic needs and sharing responsibilities with the mother, such as caring for other children and household chores. Emotionally, fathers promote the development of an emotional bond between mother and baby and participate in caring for the child by providing emotional support to the mother-baby dyad^(8)^.

In a systematic review aimed at analyzing parental interventions for promoting infant development in children under 3 years old, it was observed that the majority of interventions (93%) focused exclusively on mothers, while 7% involved fathers to some extent. Even with studies included from 33 different countries, mostly high-income, fathers are still minimally involved in actions to promote infant development^(9)^.

In a comparative survey conducted among five countries (Brazil, Spain, Australia, the United States, and Portugal), it was observed that positive parenting promotion programs are mainly embedded in the context of domestic violence perpetrated by men. This understanding allows healthcare professionals to implement more effective and targeted socioemotional interventions in caring for families, which is a fundamental strategy in the field of public policies^(10)^.

In Brazil, the National Policy for Men’s Health Care seeks to promote and discuss issues related to responsible paternal parenthood and the inclusion of men in caring for their children. It aims to raise awareness among managers, healthcare professionals, and the general population about the benefits to infant development through the active involvement of men^(11)^.

The Early Childhood Legal Framework, a Brazilian federal law that established principles and guidelines for the formulation of public policies and programs for children up to 6 years of age, emphasizes the importance of the presence of a responsive caregiver who meets the needs of children. The Brazilian National Policy for Comprehensive Child and Adolescent Health Care (PNAISC) also reinforces the importance of parental presence for the comprehensive development of the child^(2,12)^.

Internationally, child development has also been a priority for authorities, and the Nurturing Care strategy, launched by the World Health Organization (WHO) in 2018 as a set of global actions aimed at responsive childcare, highlights child development (CD) as one of the pillars to achieve the Sustainable Development Goals (SDGs) by 2030^(13)^.

Among the benefits of paternal involvement in child development are security, strengthening the father-child/family bond, personality formation, ethical-moral character maturation, and better socioemotional development^(14)^.

Among the actions that promote positive parenting and strengthen parental skills are the development, by professionals in Primary Health Care, of prenatal consultations, home visits, childcare consultations, and educational groups, among others, and these should be promoted in all care settings^(15)^.

To consider caregiving actions that strengthen the exercise of parenthood developed by men, it is relevant to understand their perceptions of this role. It is noted that current studies on paternal parenthood are focused on prenatal areas and on understanding the role of men in care before the child’s birth, requiring studies that can understand men’s involvement in promoting child development^(16)^.

## OBJECTIVES

To understand men’s perception of paternal parenthood in caring for infants to promote child development.

## METHODS

### Ethical Aspects

The study was conducted in accordance with national and international ethical guidelines and approved by the Ethics Committee of *Universidade Cesumar*. Informed consent was obtained from all study participants. To preserve anonymity, participants’ names were replaced by the abbreviation “P” for Interviewed Father, followed by numbers from one to fifteen, according to the order of the interviews.

### Study Design

This is an exploratory qualitative study^(17)^. The Consolidated Criteria for Reporting Qualitative Research (COREQ) instrument^(18)^ was used to guide the methodology.

### Participant Selection and Research Setting

Participants were selected for convenience and invited to participate in the study during breaks between classes at a university in southern Brazil, after explaining the study’s objectives. Fifteen fathers participated, including faculty, students, and administrative staff at the institution. The inclusion criterion was having children aged between 6 months and 2 years. Fathers from mononuclear families were excluded to understand men’s perception of caregiving in parental activities, where the mother is also present. There were no refusals from participants.

### Data Collection and Organization

Data collection took place from February to July 2022 through semi-structured interviews, individually audio-recorded, in-person, and in a reserved space. The guiding questions for the research were: “Explain in your own words what being a father means to you”, “How do you take care of your child under 2 years old?”, “In your opinion, what actions can you perform to promote the infant development of your child under 2 years old?”, “What difficulties do you face in caring for your child under 2 years old?”, “For you, what is the importance of your participation in caring for your child under 2 years old?” and “Do you believe you can meet all your child’s needs?”. In addition to the questions, sociodemographic data collection was performed to characterize the participants. The interviews had an average duration of 10 minutes, and only one meeting was held with each participant. The data collection script was pre-tested, and this interview was not included in the presented results. The interviews were conducted by two students trained by the principal researcher, one female and one male in the final year of the Nursing course, who had no direct relationship with the interviewees. The invitation to participate in the interview was made before the start of class or during breaks. Data collection was concluded when the objectives were achieved, and the research questions were answered.

### Data Analysis

The interviews were fully transcribed and analyzed using the Content Analysis technique, in the thematic modality^(19)^. The analysis was conducted in three steps: pre-analysis, which involved organizing ideas through a thorough reading of the material, establishing hypotheses and objectives, and developing indicators to support the final interpretation; exploration of the material, which entailed coding the raw text data; and data categorization, which included data treatment, inference, and interpretation, through the isolation/differentiation of elements in Excel® spreadsheets, followed by regrouping based on similarities.

After the research was concluded, participants were invited to take part in the Nursing course’s thesis exhibition, where the research results were presented.

## RESULTS

Fifteen men participated in the study, aged between 25 and 40 years, including 9 students, 4 faculty members, and 2 administrative staff from higher education institutions. The professions mentioned by students and administrative staff, in addition to their affiliation with the data collection institution, were: Ambulance Driver (3), Nurse (3), Nursing Technician (2), Radio Operator of the Mobile Emergency Care Service - SAMU (1), Dentist (1), and Systems Analyst (1). Regarding the total number of children, 3 fathers had three children, 6 had two children, and 6 had one child. Concerning the age of the participants’ children, it ranged from 7 months to 22 years. When asked about participation in courses promoting paternal parenthood, only 2 interviewees reported having participated.

After analyzing the statements, two categories emerged: “Perception of being a father: challenges and novelties” and “Promotion of child development: actions performed by fathers”.

### Perception of Fatherhood: Challenges and Discoveries

Many fathers express feeling unprepared to confront the challenges of parenthood, encountering various obstacles. Despite these difficulties, they emphasize the importance of serving as ethical and moral role models for their children, demonstrating responsibility and dedication to parenthood. Participants describe their experience as fathers as challenging, new, and rewarding when caring for and protecting their children.

*Being a father for me is a challenge, especially in today’s world, particularly during and after the pandemic. Being a father is the toughest part, it’s about raising children. At the same time, trying to protect them and shield them from some of the things this world has is one of the greatest challenges. It’s the educational part that weighs the most.* (F8)

*Unique experience, it’s hard work but rewarding.* (F10)

*Being a father for me is very rewarding, I like it and I try to be as involved as possible in raising my daughters because they are all girls.* (F13)

*A father is someone who loves, cares, protects, provides, educates, admonishes, shows the right path, assists, and guides in life’s discoveries.* (P14)

The lack of preparation to exercise paternal parenthood was reported as difficulty in meeting the needs of their children, the social pressure of parental responsibility, and its influence on their lives:

*I try, if I can meet all* [the needs]*, then it’s already another part. Because there will always be some difficulty, but I try the best way possible.* (P2)

*Being a father was something that came unexpectedly, at a time when I didn’t expect it. At first, I thought I wouldn’t be able to take care of a being that transforms us. Because, whether we want it or not, it changes everything. I can’t explain it, it’s a unique thing, a set of feelings that we develop.* (P5)

[...] *I think there are some things that I don’t have knowledge or preparation for. It’s difficult to measure, to make something tangible, to talk about, but the child’s own growth and development often have things that go unnoticed. I think it would be impossible to handle everything one hundred percent.* (P12)

*Many times I find myself in despair, I don’t know what to do.*(P15)

In addition to the lack of preparation, the lack of time to engage in paternal parenthood was identified by fathers, as they are unable to be present due to work commitments:

*I lack time because of work. I spend some time absent, so I think that leaves a gap.* (P1)

[...] *I can’t meet all her needs because it depends on work, college, I’m rushing, studying, and everything. So I end up not dedicating all the time I wanted to dedicate to her. I would like to be a little more present, a little more active but unfortunately, I can’t respond adequately.* (P5)

*I can’t because I lack time, due to my two jobs. I can’t fulfill everything, because I should have to work less and spend more time to be able to develop this* [care] *better.* (P8)

I work a lot, only on my days off can I spend time with them. (P13)

Among the novelties, participants also report concern about being an ethical and moral example for the development of their children:

[...] *having the responsibility of taking care of a child, caring for them in every way, giving affection, educating, setting a good example, making sure they especially follow your good examples to become someone in life. To have a future.* (P1)

[...] *I think the first thing to pass on at this age is what is right and wrong. What can and cannot be done.* (P2)

[...] *The father figure and the mother figure are central in the child’s life. They set a reference for the rest of their life,* [...]. (P3)

*Being an example for someone is being careful with everything you do. Because from a simple swear word to a habit of leaving a cup lying in the sink or something like that, you end up being a mirror.* (P11)

### Promotion of child development: actions performed by fathers

Participants reported the need for emotional support, everyday caregiving actions, and also mentioned activities aimed at promoting their children’s socioemotional development. Close and affectionate care was identified as providing emotional support and fostering emotional bonds through paternal presence and demonstrations that convey care and emotional comfort to the child, such as affection and emotional reassurance:

[...] *being present, feeling that he wants my presence. I had present parents, and it won’t be any different with him. To meet all* [needs]*, I would have to be one hundred percent with him. There’s always something missing, but in the scenario I live in, I manage to meet his needs as much as possible.* (P7)

*I participate a lot, and I consider the father’s daily presence very important. My presence brings security and emotional bond.* (P14)

*I believe the most important thing is to be present, no matter the situation, whether you know what to do or not, but being there demonstrating love and showing her that you are there with her.* (P15)

The fathers also mentioned caring for the child’s health, assisting in daily activities such as feeding, bathing, medical appointments, and encouraging leisure time:

*I take care of him, give him baths, feed him, educate him, teach him, play with him, and when I need to correct, I make corrections.* (P1)

*In the moments we have to be together, as our lives are a bit rushed, I try to make the most of it. I give affection, attention, try to always be present with her, myself, change and feed her, breastfeed.* (P2)

*I take care of her. On the days when I am at home, we play, have fun. I help with feeding and some chores. I try to take care of bathing, feeding, and try to spend the free time I have with them.* (P7)

*I seek to take care of her in the healthiest way possible, always present at her pediatrician, but as she is my first and only daughter* [...] *then I turn to the advice of my parents and my wife’s parents. Since they’ve been through this several times, it makes me feel more relaxed.* (P15)

It is also noticeable that fathers seek to engage in activities that stimulate the child’s reasoning and language development, such as repeating phrases, words, and/or asking questions, as well as adopting attitudes aimed at promoting the child’s independence. Cognitive and socioemotional stimuli used by fathers include painting, drawing, memory games, online videos, reading books, using music, interacting with animals, and spending time in nature:

*Playing with him, not adhering so much to today’s modernity.* (P4)

[...] *I try to teach the basics, some letters, numbers, some things for her to try to develop through play.* (P5)

*Interaction, playing, and especially affection.* (P6)

[...] *I think what develops is the part of playful activities. The games we have at home, musical activities, and drawing.* (P8)

[...] *games, music. Now with the internet, everything becomes easier, sometimes it’s a little song, if she’s crying you just put it on, it already develops her. It’s a little drawing, although she’s not much into drawing. She prefers music, and Miguel prefers little videos. He already has the drawings he likes.* (P9)

*Encouraging talking, encouraging crawling.* (P10)

*She attends a private school, and we received some guidance on that. Introducing classical music, playful games, and talking. Even though I can’t understand much of what she says due to her age. Painting, drawing, are ways that the school has passed on to us to help her development, especially these building games. Games involving colors and fitting, these playful games are to develop, all this area related to arts.* (P11)

## DISCUSSION

The participants represent a diverse range of ages, occupations, and number of children, offering a comprehensive understanding of paternal parenthood across various life stages and professional settings. Notably, the significant presence of healthcare professionals in the study may influence their perception of infant care due to their proximity to and understanding of healthcare practices. Formal preparation for parenthood is limited, with only two fathers having participated in courses promoting paternal parenthood, suggesting that most fathers may not be adequately prepared for child care. Additionally, the majority of men hold two jobs, indicating extensive work hours and multiple responsibilities that impact the time and energy available to dedicate to their children.

Research presenting epidemiological data on men’s relationship and involvement in childcare is still scarce. In the latest report presented by Helping Dads Care^(20)^, concerning reasons why men take paternity leave, in 2018, less than 1/3 of fathers reported working in companies that did not guarantee this right. The research revealed that 64% of the sample expressed interest in extending the leave from 5 to 20 days, as established by the Company Citizen law. Regarding satisfaction with the paternity leave policy in Brazil, 87% of men and 93% of women reported being satisfied. When asked about their involvement in childcare, 37% of men said they did not have enough time to engage in activities promoting their children’s development, while 59% expressed satisfaction with their involvement with their child.

It is evident that research discussing the policy of parental leave reconciliation between childcare and men’s work is needed, as presented by Brazil’s National Plan for Early Childhood (PNPI)^(21)^.

Brazil’s PNPI is a political and technical guide that directs the protection and promotion of children’s rights in the first six years of life, emphasizing the child’s social understanding as a rights holder. Its actions encompass integration with National Plans for Education and Health, addressing the rights of children aged 0 to 6, such as family coexistence, mental health, play, and civil registration. In the latest revision, the role of men in family care was included, especially in the context of Partner Prenatal Care in Primary Care, aiming to involve men during pregnancy, along with proposals for postgraduate courses on child development and parenthood^(21)^.

Paternal parenthood is described by the fathers in this study as a challenging experience, involving adaptation to a new phase of life with increasing responsibilities and the social and personal pressures associated with fatherhood. Despite the challenges and new experiences, paternal parenthood is also portrayed as a fresh and fulfilling journey. The historical construction of gender roles varies, with men typically directed towards financial and material responsibilities, while women are often tasked with emotional demands that contribute collaboratively to children’s emotional development^(22)^. There’s a notable emphasis on the active role fathers play in daily caregiving, extending beyond merely providing sustenance or assuming an authoritarian role. Care encompasses various aspects of a child’s development and well-being, illustrated by routine tasks like feeding and attending medical appointments.

It’s evident that despite legislative efforts in Brazil, the number of effective policies encouraging paternal parenthood remains low, potentially impacting fathers’ presence and involvement in childcare during a child’s early years. Public policies are continually under discussion to better align with the needs of paternal practices. Currently, Brazilian policies promoting paternal parenthood include Paternity Leave, laws facilitating support for pregnant and postpartum women, Adoptive Leave, the Statute of the Child and Adolescent, and the National Policy for Comprehensive Men’s Health Care (PNAISH)^(2)^. However, there remains a scientific gap in legal initiatives promoting paternal parenthood, hindering theoretical exploration on the topic and child development, despite the existence of the National Plan for Early Childhood since 2010.

In addition to physical and practical care, paternal parenthood encompasses emotional aspects, involving the demonstration of affection, love, understanding, and other forms of emotional expression. Public policies and employers play pivotal roles in promoting a better balance between career and parenthood. Stereotypes of hegemonic masculinity and the ideal worker, prioritizing work over other responsibilities, can negatively impact the equilibrium between professionalism and parenthood, impeding men from adopting more realistic and nurturing parenting approaches^(23)^.

The latest report from the United Nations International Children’s Emergency Fund - UNICEF^(24)^ sheds light on the global status of parental leave. Until 2015, 48% of countries worldwide did not offer paid parental leave, including maternity leave. The report indicates that 68% of high-income countries provide paid leave for both parents, compared to 38% and 47% in low and middle-income countries, respectively. While 22% of countries offer at least 4 weeks of parental leave, only 3% allow the transfer of leave to the father in the absence of the mother, often not as a choice by the couple.

Under these circumstances, it’s crucial to underscore the necessity of specific public policies for paternal parenthood to drive cultural shifts and facilitate conditions conducive to child development. Regional initiatives proposing enhancements to parental practices are also essential. One noteworthy initiative is the MenCare project, a global effort aimed at broadening discussions on gender equality and encouraging men’s engagement in childcare, thereby fostering social change^(20)^.

The participants in the study describe everyday actions such as feeding, bathing, and playing, which are essential aspects of paternal parenthood. However, it should be acknowledged that childcare is still closely associated with the maternal figure. It is emphasized that emotional involvement between father and child is crucial for the child’s growth and development, and that the individual’s identity is established in childhood, shaping their moral and ethical references^(25)^.

Paternal involvement is understood to be directly linked to the level of attachment established with the child. Research indicates that fathers with secure attachment are more significantly involved in caregiving compared to those with insecure attachment. Fathers with secure attachment are able to encourage their children to engage in challenging activities, such as bathing and taking them to school^(25)^.

A systematic review^(6)^ has found that paternal involvement directly influences verbal stimulation, care, and physical interaction with the child. Regarding paternal education level, fathers with a university education demonstrate greater engagement in verbal activities, while the presence of marital conflict is correlated with lesser participation in caregiving responsibilities and physical activities with the children.

Due to the period of biopsychosocial construction and maturation, it is necessary to provide children with good conditions to prevent future socioemotional and intellectual dysfunctions, ensuring a balanced development of self-confidence. An individual with maturity will adequately perform autonomy and establish positive interpersonal relationships^(26)^.

The environment should provide an experience based on emotional comfort through the sensitivity of the father and/or caregiver during childhood, promoting attachment and contributing to meeting the child’s needs. There is also a need to assist in conflict resolution to promote the development of social and emotional skills, alongside self-regulation defining self-perception, providing an active and quality presence between father and child^(26)^.

To improve this scenario, WHO has developed a handbook for parents, which aims to guide responsibilities and actions to fulfill parenthood. Thus, the importance of paternal parenthood is reinforced to promote actions that go beyond physical care but also involve emotional involvement, ensuring greater emotional bond and promoting child development^(27)^.

The lack of skills among men, coupled with insecurity about their ability to interact with children, suggests a lack of guidance in Primary Health Care^(28)^. However, it is observed that there is still little interest from men in learning about newborn care, further emphasizing the need for guidance, discussions, and an active search for a better understanding of the importance of their preparation and active participation^(29)^.

The role of nurses in prenatal and childcare consultations is crucial in inviting fathers to participate in consultations and involving them in care. It is important for nurses to recognize the father’s importance for the child’s development and, therefore, encourage their participation from prenatal consultations, including offering guidance and training so that they feel more capable of conducting care within the family, at home.

A study aimed at describing paternal competence, based on guidance provided by the nursing team, identified that fathers of newborns who received instructions felt more prepared and adequately instructed to provide care to the baby. These guidelines enabled them to understand the needs to be met in each period of their child’s early life, intervening and acting with confidence, providing positive experiences and emotions between father and child, thus ensuring the child’s healthy growth and development^(30)^.

Thus, paternal parenthood is understood as a dynamic and continuous process that occurs through the father’s relationship with his family and himself, ensuring a better quality of life and healthy emotional bonds^(31)^. In this research, the lack of time to engage in paternal parenthood was mentioned as commonplace, given the daily demands of balancing work, studies, and childcare. Balancing career and parenthood seems to be a real obstacle in the father’s life, as public policies that can support this social and cultural progress are still scarce, hindering the achievement of gender equality^(32)^.

Parents recognize the importance of activities that promote their children’s overall development. Among the mentioned actions are cognitive and socio-emotional stimuli that provide a basis for child development. Research has shown the importance of the role of fathers in promoting their children’s cognitive and socio-emotional development. The active presence of fathers in children’s daily lives increases their learning and stimulation opportunities. One of the tools that parents frequently use to enrich their children’s educational experience is music^(6)^. Exposure to music from an early age has the potential to improve children’s cognition and social skills. Moreover, activities such as drawing, playing, and interacting with nature provide multiple benefits, helping to develop fine motor skills and stimulate creativity^(33)^.

Parents, when speaking and actively responding to the sounds emitted by their children, encourage speech development. In addition to language, stimulating movements, such as crawling, is essential for motor development^(34)^. Playful interactions, such as helping the baby move on different surfaces, can accelerate this process^(35)^.

Playful games are not only a source of fun for children but also a powerful learning tool. Play, especially imaginative and unstructured play, promotes children’s cognitive, emotional, and social development. Furthermore, parents who engage in practical play, such as building blocks or puzzles with their children, provide valuable opportunities for spatial thinking development and problem-solving skills^(36)^.

The necessity of the paternal figure during development is evident, and this can be effectively achieved in various ways, such as through play, as described by the participants. Play is as crucial for a child as their basic needs for feeding and sleeping, as it allows children to explore, learn social rules, and deepen their development^(37)^. Play experiences encompass different age groups and can include games, toys, and activities. These forms of entertainment serve as a learning tool through which dreams, fears, fantasies, and frustrations can be expressed^(38)^.

Play awakens various internal developmental processes in the child and works when the child interacts with people in diverse environments and cooperates with peers. Thus, play plays an important role in learning and social integration, as it arouses emotion and imagination, giving shape and density to the experience of perceiving, feeling, and thinking, creating internal images that combine to represent that experience. Imagination is the root of any intellectual process, whether scientific, artistic, or technological. These early social interactions, forged in the context of play, are fundamental to the formation of the individual and, consequently, to their understanding and practice of morality and ethics^(39)^.

Interaction with nature, mentioned by parents as one of the strategies for promoting child development, has been discussed by some studies as a necessary and fundamental action for child development. Outdoor ecological experiences promote, in addition to emotional involvement between parents and the exploration of new places, contribute to more sustainable behaviors. In addition to influencing environmental concern and future behavior towards the environment, promoting play in nature contributes to the conservation of biodiversity^(40)^.

The construction of paternal parenthood is a process constructed and experienced individually and is influenced by multisystemic factors. Among the factors considered fundamental for the promotion of paternal parenthood, we can mention: men’s individual health, the learning process about parenthood, socioeconomic aspects, social support for promoting positive interactions, legislative and political support to foster parenthood, as well as the preservation of cultural continuity. These elements are essential to foster and promote fathers’ involvement in raising children^(6)^.

### Study limitations

This study has some limitations, including the inability to generalize the data as representative of Brazilian fathers, since the results refer to the individual experience of men associated with the academic environment and mostly linked to the healthcare sector, which may not reflect the diversity of experiences of paternal parenthood in various socioeconomic and cultural contexts in Brazil. Additionally, the lack of a more in-depth exploration of the underlying causes of the time shortage reported by the participants constitutes a gap in the scope of the research.

### Contributions to Health and Public Policy

The study offers a comprehensive view of paternal parenthood at different stages of life and professional contexts. It also highlights the lack of formal preparation for the practice of paternal parenthood. In the context of public policies, it is possible to emphasize that the participants recognize the challenges, lack of preparation, and time constraints as obstacles to paternal parenthood. Their actions aim to promote the comprehensive development of their children, including cognitive, socio-emotional strategies, and interaction with nature. The lack of specific policies and the need for guidance are discussed, highlighting the importance of social understanding and the role of healthcare professionals in promoting paternal parenthood. The involvement of fathers in activities that promote the comprehensive development of children, such as cognitive stimuli, playful games, artistic activities, and emotional interactions, is emphasized. These actions are fundamental for the cognitive, emotional, and social development of children, contributing to healthy growth and strengthening family bonds.

## FINAL CONSIDERATIONS

It is understood that the experience of paternal parenthood is described as challenging and rewarding, being essential for the care and protection of children. The lack of preparation and time constraints are recognized as significant challenges for men’s parental roles. Among the actions that promote child development, men highlight the importance of activities that enhance cognitive and socio-emotional development, such as games, music, art, and interaction with nature. The role of healthcare professionals, especially nurses, is crucial in guiding and involving men in childcare, from prenatal care. This involvement can result in better-prepared and more empathetic fathers to positively stimulate child development.

This study emphasizes the need for public policies that support paternal parenthood, emphasizing the importance of participation in prenatal and childcare consultations, and the need to provide an emotionally safe environment for the child. Future studies could further explore the long-term effects of paternal parenthood on child development, exploring areas such as balancing career and parental responsibilities.

## Supplementary Material

0034-7167-reben-77-03-e20230514-suppl01

## Data Availability

https://doi.org/10.48331/scielodata.OKGAXW
