# Supplementary material for: Men’s perception of paternal parenthood and the promotion of child development
Source: Rev Bras Enferm. 2024 Jul 29;77(3):e20230514. doi: 10.1590/0034-7167-2023-0514 (PMC11290730; doi:10.1590/0034-7167-2023-0514)
Supplement: 0034-7167-reben-77-03-e20230514-suppl01 [file 0034-7167-reben-77-03-e20230514-suppl01.pdf]

DATA:09/08/2022 ( ) DOCENTE ( ) COLABORADOR (x) ESTUDANTE

**DADOS DE IDENTIFICAÇÃO**

CÓDIGO DE IDENTIFICAÇÃO: P1

IDADE: 38 anos

ÁREA DE ATUAÇÃO: Condutor de Ambulância

NÚMERO DE FILHOS: 2 IDADE DOS FILHOS: uma menina de 12 anos e um menino de 1 anos e 10 meses.

QUANTOS VÍNCULOS EMPREGATÍCIOS POSSUI ALÉM DA ATIVIDADE NA UNIVERSIDADE? 2

REALIZOU CURSOS DE PATERNIDADE ANTES DO NASCIMENTO DE SEU FILHO? Não.

FREQUENTA HÁ QUANTO TEMPO O ÂMBITO UNIVERSITÁRIO? 2 anos.

**QUESTÕES DA PESQUISA**

1) Explique com suas palavras o que significa ser pai para você.

   Ah, o que significa e você ter a responsabilidade de cuidar de uma criança, cuidar de todas as formas, tipo dando afeto, educando, dando bom exemplo, fazendo com que principalmente siga teus bons exemplos, pra não cair, como se diz assim, pra ser alguém na vida pra ter futuro.

2) Como você cuida de seu filho com menos de 2 anos?

   Ah, eu cuido, dou banho, alimento, educo, ensino, brinco com ele quando preciso corrigir faço as correções.

3) Na sua opinião, quais ações que realiza podem promover o desenvolvimento infantil de seu filho menor de 02 anos?

   As ações são brincadeiras, as orientações, os cuidados.

4) Quais dificuldades você enfrenta no cuidado com o seu filho com menos de 2 anos?

   Eu acho que o principal e a ausência, falta de tempo por causa dos dois trabalhos, ai eu passo um tempo ausente assim, acho que isso e o principal.

5) Para você, qual é a importância da sua participação no cuidado do seu filho menor de 2 anos?

   Ah, eu acho que é a importância e grande assim, porque tem a minha esposa mas, eu também ajudo nessa parte de cuidados tudo, de brincadeiras.

6) Você acredita que consegue responder todas as necessidades de seu filho?

   Acho que não, porque justamente por essa falta de tempo, por causa dos trabalhos tudo eu passo um tempo ausente, então acho que isso deixa uma lacuna.

DATA: 09/08/2022 ( ) DOCENTE (X) COLABORADOR ( ) ESTUDANTE

DADOS DE IDENTIFICAÇÃO

CÓDIGO DE IDENTIFICAÇÃO: P2

IDADE: 39

ÁREA DE ATUAÇÃO: Enfermeiro.

NÚMERO DE FILHOS: 3 IDADE DOS FILHOS: uma de 1 ano, tem um de sete e um de 13.

QUANTOS VÍNCULOS EMPREGATÍCIOS POSSUI ALÉM DA ATIVIDADE NA UNIVERSIDADE? 2 vínculos.

REALIZOU CURSOS DE PATERNIDADE ANTES DO NASCIMENTO DE SEU FILHO? Realizou um.

FREQUENTA HÁ QUANTO TEMPO O ÂMBITO UNIVERSITÁRIO? 6 a 7 anos.

QUESTÕES DA PESQUISA

1) Explique com suas palavras o que significa ser pai para você.

   Ser pai pra min, eu tive que na verdade me redescobrir, porque eu tinha dois meninos e veio uma menina, então eu tive que reaprender a ser pai de menina, ta sendo uma experiência maravilhosa pra min, tudo novo, e diferente, ta sendo muito bom essa minha fase com minha filha, consegui ter um pouco mais de tempo com ela do que eu tive com os outros dois, então pra min está sendo maravilho esta experiência.

2) Como você cuida de seu filho com menos de 2 anos?

   A gente tenta, nos momentos que a gente tem pra ficar junto, como a nossa vida e meio corrida né, a gente tenta aproveitar da melhor maneira possível, do carinho da atenção, tento estar sempre presente com ela, eu mesmo, troco dou alimentação, da mama, então a gente tenta ta, aproveitar o maior tempo quando a gente está junto possível.

3) Na sua opinião, quais ações que realiza podem promover o desenvolvimento infantil de seu filho menor de 02 anos?

   Olha a gente tenta passar, desde essa idade ai, as coisas certas, a gente sabe que criança, ta sempre, se dar um descuido pode sofrer um acidente,

então a gente, primeira coisa que a gente tenta ensinar nessa idade e a andar, e a primeira coisa que a gente tenta ensinar e a andar, tenta passar pra eles, a gente quer que eles andem o mais rápido possível, então eu acho que a primeira coisa tenta fazer e passar pra eles e de como eles se virar, como eles são um pouquinho mais independente nisso ai na parte de gatinhar, andar, a minha ainda está na fase de ainda de aprender a gatinhar, andar, a gente tem um cuidado, eu acho que a primeira coisa a passar nessa idade, e o que é certo e errado, o que não pode colocar na boca, o que pode o que não pode.

4) Quais dificuldades você enfrenta no cuidado com o seu filho com menos de 2 anos?

   Eu acho que, a maior dificuldade que a gente tem hoje, como pai e mãe trabalha, deixar com a baba, acontece de vez em quando a baba ligar para você e falar que ela não está bem, está com febre você tem, ou eu ou a mãe dela sair correndo do serviço, deixar trabalho de lado, e vir correndo pra casa, pra poder levar no medico, acredito eu que a maior dificuldade nossa hoje, a gente passou por algumas experiências ai meio ruins então, eu acho que dificuldade a gente tem nesse período pra falar , a nossa maior dificuldade está sendo isso.

5) Para você, qual é a importância da sua participação no cuidado do seu filho menor de 2 anos?

   Eu acho que, importância total, pra ela ter o pai dela do lado assim, podendo estar ali junto com ela, ta ensinando gatinhar, ensinando andar, falar as primeiras palavras, eu acho que, não tem assim e de suma importância pra ela assim, ter esse vínculo comigo, uma coisa acredito eu que ela vai levar pro resto da vida, tento o pai dela ali ensinando pra ela.

6) Você acredita que consegue responder todas as necessidades de seu filho?

   Eu assim, a gente tenta né, se vai conseguir sanar todas ai já e uma outra parte porque sempre vai ter alguma dificuldade, então nem sempre a gente consegue resolver da maneira que deveria mas a gente tenta da melhor forma possível.

DATA: 06/08/2022 ( ) DOCENTE (X) COLABORADOR ( ) ESTUDANTE

**DADOS DE IDENTIFICAÇÃO**

CÓDIGO DE IDENTIFICAÇÃO: P3

IDADE: 37 anos

ÁREA DE ATUAÇÃO: Dentista

NÚMERO DE FILHOS: 2 IDADE DOS FILHOS: ester 2anos e 3 meses e o Pedro 12 anos

QUANTOS VÍNCULOS EMPREGATÍCIOS POSSUI ALÉM DA ATIVIDADE NA UNIVERSIDADE? 2

REALIZOU CURSOS DE PATERNIDADE ANTES DO NASCIMENTO DE SEU FILHO? Não

FREQUENTA HÁ QUANTO TEMPO O ÂMBITO UNIVERSITÁRIO? 7 anos

**QUESTÕES DA PESQUISA**

1) Explique com suas palavras o que significa ser pai para você.
   Ah e uma grande responsabilidade pra gente ter uma pessoa nossa sobre nossa tutela sobre nossa proteção e também um incentivo pra gente melhorar como pessoa como ser humano pelo exemplo.
2) Como você cuida de seu filho com menos de 2 anos?
   Todos os cuidados gerais desde educação até o banho, comida, os a gente tenta a parte de desenvolvimento ensinar ela falar melhor, ela escrever, ela aprender alguma coisa nova, brincadeira, as partes não deixo só a cargo da minha esposa.
3) Na sua opinião, quais ações que realiza podem promover o desenvolvimento infantil de seu filho menor de 02 anos?
   E principalmente através das brincadeiras assim do modo lúdico pra gente poder deixar o desenvolvimento mais fácil ne, sem ser uma coisa maçante A gente pode fazer brincadeiras para memorização pro conhecimento de letras, números, outras línguas, tudo esse tipo de coisa lúdica assim.
4) Quais dificuldades você enfrenta no cuidado com o seu filho com menos de 2 anos?

A gente sempre fica se cobrando na parte em que a gente pode fazer de melhor no pra não também forcar demais a criança, minha preocupação maior e não deixar ela, como eu posso te dizer, e realmente forcar, forçar, deixar muito forcado essas atividades que a gente impõem para as crianças, deixar ela ter uma naturalidade pra ela brincar, pra ela ter o tempo dela, eu acho que falta as vezes um pouco também de ter uma orientação melhor assim sobre isso sabe, uma coisa, um jeito mais fácil de buscar conhecimento sobre como estimular a criança.

5) Para você, qual é a importância da sua participação no cuidado do seu filho menor de 2 anos?

   Ah e total né, figura paterna e a figura materna são centrais na vida da criança né, eles fazem uma referência para o resto da vida dela, ela sempre baseia as coisas no pai na mãe, até nas brincadeiras dela.

6) Você acredita que consegue responder todas as necessidades de seu filho?

   Olhe, não sei, acho que essa e a grande dúvida dos pais. A gente sempre busca melhorar a cada dia assim, sempre observar o comportamento, sempre tentar buscar perguntar pra eles como estão se sentindo mas não sei a gente nunca sabe, principalmente pela parte emocional né, como eles estão se sentindo verdadeiramente, mas e uma busca constante, por atingir esse objetivo de melhorar a vida da criança, ter ela menos difícil.

DATA: 24/07/2022 ( ) DOCENTE ( ) COLABORADOR (x) ESTUDANTE

DADOS DE IDENTIFICAÇÃO

CÓDIGO DE IDENTIFICAÇÃO: P4

IDADE: 26 anos

ÁREA DE ATUAÇÃO: Analista de Sistemas

NÚMERO DE FILHOS: 1 IDADE DOS FILHOS: 10 meses

QUANTOS VÍNCULOS EMPREGATÍCIOS POSSUI ALÉM DA ATIVIDADE NA UNIVERSIDADE? Somente um mesmo.

REALIZOU CURSOS DE PATERNIDADE ANTES DO NASCIMENTO DE SEU FILHO? Não.

FREQUENTA HÁ QUANTO TEMPO O ÂMBITO UNIVERSITÁRIO? Desde 2017.

QUESTÕES DA PESQUISA

1) Explique com suas palavras o que significa ser pai para você.

   Ah uma coisa diferente né, e uma coisa nova pra min agora, então mudou totalmente a rotina, tanto minha quanto da mãe dele, minha mulher, são vários desafios que a gente e que ela tem 29 anos, não é tão nova sim, já estava na idade de ter filhos mas, e uma coisa que mudou bastante a nossa rotina mas ser pai ta sendo muito bom ta sendo legal tem suas partes difíceis mas a maior parte e boa.

2) Como você cuida de seu filho com menos de 2 anos?

   No dia, como eu trabalho uma noite sim outra não, 12/36, a noite a mãe que fica, nessa noite que eu trabalho, ela fica em casa la cuidando, fica cuidando, e na outra noite eu e ela que cuidados e durante o dia sou eu que cuido porque ela trabalha durante o dia.

3) Na sua opinião, quais ações que realiza podem promover o desenvolvimento infantil de seu filho menor de 02 anos?

   Brincar né com ele, não aderir tanto modernidade de hoje em dia que e muita televisão, celular na mão de criança, fazia a brincadeira de como era antigamente.

4) Quais dificuldades você enfrenta no cuidado com o seu filho com menos de 2 anos?

   Essa questão mesmo do trabalho, porque, essa questão de, da minha parte, descanso, que uma noite eu estou trabalhando, durante o dia estou cuidando dele, e não outra noite mãe está junto mas ela também tem que descansar porque ela trabalha durante o dia, então essa questão do meu descanso e complicado, fica meio difícil.

5) Para você, qual é a importância da sua participação no cuidado do seu filho menor de 2 anos?

   Eu acho que isso ai vai refletir bastante no futuro dele, porque a gente vê muitas situações ai que acontecem geralmente porque a pessoa não tem a presença do pai presença masculina ali na vida dele, não só até os 2 anos mas como durante a vida inteira, existe algumas situações que as vezes acontecem devido essa falta do pai.

6) Você acredita que consegue responder todas as necessidades de seu filho? Acredito que sim, até agora sim.

DATA: 24/07/2022 ( ) DOCENTE ( ) COLABORADOR (x) ESTUDANTE

DADOS DE IDENTIFICAÇÃO

CÓDIGO DE IDENTIFICAÇÃO: P5

IDADE: 28 anos

ÁREA DE ATUAÇÃO: Condutor de Ambulância

NÚMERO DE FILHOS: uma filha. IDADE DOS FILHOS: 2 anos.

QUANTOS VÍNCULOS EMPREGATÍCIOS POSSUI ALÉM DA ATIVIDADE NA UNIVERSIDADE? Além, tenho dois.

REALIZOU CURSOS DE PATERNIDADE ANTES DO NASCIMENTO DE SEU FILHO? Não.

FREQUENTA HÁ QUANTO TEMPO O ÂMBITO UNIVERSITÁRIO? 3 anos.

QUESTÕES DA PESQUISA

1) Explique com suas palavras o que significa ser pai para você.
   Cara, pra min, ser pai foi uma coisa que veio assim num momento assim que eu não esperava, pra min achei de início achei que não iria se capaz de cuidar de um ser assim que transforma a gente, querendo ou não a gente muda totalmente uma coisa que a gente não espera pra min ser pai, significa muito e num não tenho certamente assim como explicar e uma coisa única e um conjunto de sentimentos que a gente desenvolve.
2) Como você cuida de seu filho com menos de 2 anos?
   Então, e normalmente eu deixo ela mais a vontade, tipo ela saber tudo no tempinho dela, não tem como cobrar de uma criança uma coisa que ela ainda não entende, então pra min a gente mantém ela mais assim, ela tem um probleminha renal, então sempre mais cuidando dessa parte, devido esse probleminha, a gente deixa ela brincar a vontade, ter os horários dela mais à vontade, não prender muito não querer ficar forcando muito a criança.
3) Na sua opinião, quais ações que realiza podem promover o desenvolvimento infantil de seu filho menor de 02 anos?

Normalmente eu sento junto com ela, a gente tenta ir ensinando o básico, tipo alguma coisinha, algumas letras números algumas coisas para ela tentar ir desenvolvendo essa parte assim tudo de forma de brincadeira para ela se interessar mais, tipo jogo de memória algumas coisinhas assim também.

4) Quais dificuldades você enfrenta no cuidado com o seu filho com menos de 2 anos?

   Então, dificuldades nessa parte, a gente tem um pouco mais de dificuldade, porque dos probleminhas renais dela, sempre tem que estar fazendo acompanhamento e também no caso algumas partes dos horários devido a faculdade correria do trabalho então um pouco assim.

5) Para você, qual é a importância da sua participação no cuidado do seu filho menor de 2 anos?

   A cara, pra um filho crescer sem pai, eu acho que deve ser a pior coisa do mundo, então assim eu tento fazer por ela o que a gente quase não teve na infância, quando o meu pai era um pouco mais ausente, então assim eu pretendo, depois conforme fui crescendo eu senti muito a falta da presença paterna então assim eu imagino que pra min estando um pouco mais presente na vida dela seria mais ideal ajuda no desenvolvimento tanto familiar entre o laço, entre o pai e o filho no desenvolvimento.

6) Você acredita que consegue responder todas as necessidades de seu filho?

   Então, eu acredito que não, não consigo atender todas as necessidades dela porque assim, a gente depende tanto do serviço da faculdade ta correndo estudando e tudo, então assim acabo não dedicando todo o tempo que eu queria me dedicar a ela, então assim eu gostaria de ser um pouco mais presente, um pouco mais ativo assim na vida dela mais infelizmente, não consigo responder a altura.

DATA: 13/04/2022 (x) DOCENTE ( ) COLABORADOR ( ) ESTUDANTE

**DADOS DE IDENTIFICAÇÃO**

CÓDIGO DE IDENTIFICAÇÃO: P6

IDADE: 36

ÁREA DE ATUAÇÃO: Professor

NÚMERO DE FILHOS: 1 IDADE DOS FILHOS: 2 anos

QUANTOS VÍNCULOS EMPREGATÍCIOS POSSUI ALÉM DA ATIVIDADE NA UNIVERSIDADE? 5

REALIZOU CURSOS DE PATERNIDADE ANTES DO NASCIMENTO DE SEU FILHO? Já, três junto com a mãe.

FREQUENTA HÁ QUANTO TEMPO O ÂMBITO UNIVERSITÁRIO? Desde 2016.

**QUESTÕES DA PESQUISA**

1) Explique com suas palavras o que significa ser pai para você.
   Ser pai e ser presente.
2) Como você cuida de seu filho com menos de 2 anos?
   Afeto cuidado disciplina e estar presente.
3) Na sua opinião, quais ações que realiza podem promover o desenvolvimento infantil de seu filho menor de 02 anos?
   Interação brincar principalmente e afeto.
4) Quais dificuldades você enfrenta no cuidado com o seu filho com menos de 2 anos?
   Tempo, presença.
5) Para você, qual é a importância da sua participação no cuidado do seu filho menor de 2 anos?
   Total o máximo que eu puder, estar presente.
6) Você acredita que consegue responder todas as necessidades de seu filho? Não, deficiente em estar presente, esposa 100% presencial eu 30%, devido rotina de trabalho e estudos.

DATA: 11/07/2022 (x) DOCENTE ( ) COLABORADOR ( ) ESTUDANTE

DADOS DE IDENTIFICAÇÃO

CÓDIGO DE IDENTIFICAÇÃO: P7

IDADE: 33 anos

ÁREA DE ATUAÇÃO: docência em educação física.

NÚMERO DE FILHOS: 1 IDADE DOS FILHOS: 1 ano e 9 meses.

QUANTOS VÍNCULOS EMPREGATÍCIOS POSSUI ALÉM DA ATIVIDADE NA UNIVERSIDADE? 1 somente.

REALIZOU CURSOS DE PATERNIDADE ANTES DO NASCIMENTO DE SEU FILHO? Curso não, somente da igreja batizado, preparação não.

FREQUENTA HÁ QUANTO TEMPO O ÂMBITO UNIVERSITÁRIO? Desde 2018.

QUESTÕES DA PESQUISA

1) Explique com suas palavras o que significa ser pai para você.

   Muita coisa, uma loucura, uma bagunça, uma mudança muito grande, antes de ser pai muitos pais iam me falar assim e cansativo muito trabalhoso mas vale a pena, escutei muito isso e prometi a min mesmo que nunca ia falar isso, só que se hoje me perguntam eu falo e cansativo, trabalhoso mas vale a pena mas e de fato ainda mais que agrego algumas questões profissionais pessoais.

2) Como você cuida de seu filho com menos de 2 anos?

   Optei, por tem um emprego esse ano para estar mais presente e pois somos uma dupla eu e minha esposa nos cuidados, pois ela trabalha em horário comercial, eu de manhã e à noite, minha sogra fica com ele no período da manhã.

3) Na sua opinião, quais ações que realiza podem promover o desenvolvimento infantil de seu filho menor de 02 anos?

   Devido eu seu pai de primeira viagem, ser pai, tudo que ele faz pra min e maravilhoso, tudo que ele faz, fala, desenvolve, tudo parece uma

novidade mais pra min do que pra ele, então todo processo de evolução de gatinhar, falar de aprender coisas de falar coisas, tudo tem sido na parte técnica tudo padrão, dentro das medias e normas e questões que não são tecnicamente e um aprendizado muito grande com ele, ele já anda sozinho, anda demais, corre , fala de tudo.

4) Quais dificuldades você enfrenta no cuidado com o seu filho com menos de 2 anos?

   Por enquanto ele absorve tudo não tem muito segredo, tentamos estimula ló muito, dificuldade tivemos no comecinho na amamentação dificuldade pelo pouco leite e sucção mas fora isso sem muitas dificuldades, e outra e a falta de tempo com ele, diminuir rotina de trabalho.

5) Para você, qual é a importância da sua participação no cuidado do seu filho menor de 2 anos?

   Ensinando passando alguma coisa, brincando, interagindo, fazemos leitura com ele.

6) Você acredita que consegue responder todas as necessidades de seu filho? Nesse processo de estimular o máximo possível, estar presente, sentir que quer minha presença, tive pais presente, e com ele não vai diferente, pra suprir todas eu estaria que estar cem por cento ele, alguma coisa sempre, sempre falta alguma coisa, mas no cenário que convivo consigo suprir o máximo que ele necessita.

DATA: 21/07/2022 ( ) DOCENTE ( ) COLABORADOR (x) ESTUDANTE

**DADOS DE IDENTIFICAÇÃO**

CÓDIGO DE IDENTIFICAÇÃO: P8

IDADE: 41

ÁREA DE ATUAÇÃO: Técnico em Enfermagem

NÚMERO DE FILHOS: 2 IDADE DOS FILHOS: 7 meses e 6 anos

QUANTOS VÍNCULOS EMPREGATÍCIOS POSSUI ALÉM DA ATIVIDADE NA UNIVERSIDADE? 2 vínculos.

REALIZOU CURSOS DE PATERNIDADE ANTES DO NASCIMENTO DE SEU FILHO? Não

FREQUENTA HÁ QUANTO TEMPO O ÂMBITO UNIVERSITÁRIO? 4 anos e 6 meses.

**QUESTÕES DA PESQUISA**

1) Explique com suas palavras o que significa ser pai para você.
   Ser pai pra min e um desafio principalmente na atualidade e nos dias que estamos vivendo principalmente durante e pós pandemia, pra min ser pai a parte mais difícil e a parte educacional, educar filhos e ao mesmo tempo tentar protege-los e blinda-los de algumas coisas que esse mundo tem e pra min esse e um dos maiores desafios mesmo e a parte de educação e a que mais pesa.
2) Como você cuida de seu filho com menos de 2 anos?
   Eu cuido dela nos dias que eu estou em casa a gente tem brinca se diverte ali junto com ela ajuda na alimentação nos cuidados alguns afazeres eu procuro trazer pra min o banho alimentação tentar gastar esse tempo que eu tenho livre com elas e com a pequenininha também.
3) Na sua opinião, quais ações que realiza podem promover o desenvolvimento infantil de seu filho menor de 02 anos?
   As que eu realizo, olha eu acho que o que desenvolve e a parte de brincadeiras lúdicas partes de brincadeiras que a gente tem em casa, musicalização e desenho não sou adepto de celular e essas tecnologias

4) Quais dificuldades você enfrenta no cuidado com o seu filho com menos de 2 anos?

   No cuidado com ela, a parte de educação mesmo de medo de a minha dificuldade e medo de errar assim na parte de educação.

5) Para você, qual é a importância da sua participação no cuidado do seu filho menor de 2 anos?

   No desenvolvimento de caráter e personalidade dela e acho que e isso.

6) Você acredita que consegue responder todas as necessidades de seu filho?

   Não, não consigo, não consigo porque me falta tempo devido aos meus dois vínculos então assim eu não consigo suprir tudo isso porque eu deveria teria que trabalhar menos e passar mais tempo para que eu conseguisse desenvolver isso melhor sabe.

DATA:21/07/2022 ( ) DOCENTE ( ) COLABORADOR (x) ESTUDANTE

DADOS DE IDENTIFICAÇÃO

CÓDIGO DE IDENTIFICAÇÃO: P9

IDADE: 38

ÁREA DE ATUAÇÃO: Radio Operador do SAMU

NÚMERO DE FILHOS: 2 filhos IDADE DOS FILHOS: O Miguel com 2 anos e 8 meses e a Helena com 1 ano e 6 meses.

QUANTOS VÍNCULOS EMPREGATÍCIOS POSSUI ALÉM DA ATIVIDADE NA UNIVERSIDADE? Eu sou funcionário Público.

REALIZOU CURSOS DE PATERNIDADE ANTES DO NASCIMENTO DE SEU FILHO? Foi pesquisando pela internet que eu fiz ali e tive um tipo assim um conhecimento pelo hospital, pela maternidade onde eles nasceram.

FREQUENTA HÁ QUANTO TEMPO O ÂMBITO UNIVERSITÁRIO? 3 anos.

QUESTÕES DA PESQUISA

1) Explique com suas palavras o que significa ser pai para você.

   Então ser pai, ali e assim como meus filhos ali eu tento da e fazer o melhor, como posso te dizer assim e tento ser a melhor pessoa ali tentando como eles são pequenos e não entendem muitas coisas assim mas a gente tenta dá o melhor assim não faltar nada sempre estar presente como eles são pequenos e a companhia ne agora, eu como pai noto assim eles gostam da companhia brincar assim, tipo eu tento fazer o meu melhor assim pra estar com eles ali.

2) Como você cuida de seu filho com menos de 2 anos?

   Então, a bichinha e terrível mas assim, em casa ali e o leite e a troca e a brincadeira, interagindo ela, como ela ta num grau tipo assim agora pequenininho de desenvolvimento assim eu tento fazer brincadeiras com elas passar o dia com ela também, não só com ela como tem o piazinho que e pequeno também, tenho que estar com os dois, com ela a gente brinca de um jeito e com ele que e maiorzinho tenta brincar de outro mas sempre mantendo os dois juntos ali e assim ne como pai ali já sabe ne ,

que onde tipo ali, eu que nem vejo pelo Miguel ali ele já papai papai não sei, o que, brincar vamos ali ele já traz os brinquedinhos vo to brincando com ele.

3) Na sua opinião, quais ações que realiza podem promover o desenvolvimento infantil de seu filho menor de 02 anos?

   Ações que, tipo posso te dizer de ações, você pode repetir a pergunta, brincadeiras, e tudo ali músicas a gente tenta agora com a internet tudo fica mais fácil ne agora alias as vezes e uma musiquinha se que ta chorando ali se já coloca ali já desenvolve ela bastante e um desenhinho apesar que ela não e muito de desenho assim ela gosta mais da música já o Miguel já gosta mais dos vidiozinho, eles já têm os desenhos que gosta.

4) Quais dificuldades você enfrenta no cuidado com o seu filho com menos de 2 anos?

   Dificuldade, então ali que eu posso te dizer, a sempre tem as dificuldades ne mas assim a gente tenta levar mas assim as dificuldades, que eu possa assim é, na doença.

5) Para você, qual é a importância da sua participação no cuidado do seu filho menor de 2 anos?

   Minha participação ali, sou o pai ne, tento fazer o melhor pra ela ali e assim e que nem eu disse ela e pequenininha ela não entende ela ta começando a aprender agora então tudo pra ela ali e brincadeira ne as vezes, as vezes assim a gente tenta fazer as coisas certas pra ela ir nas coisas certas como ela e pequenininha ainda mas acho que ela tudo pra ela e festa e brincadeira assim.

6) Você acredita que consegue responder todas as necessidades de seu filho?

   Olhe, acho que todas ali não sei te dizer assim, mas eu tento dar o meu melhor assim ne tipo quando em casa ali tudo ali a gente sabe como nosso dia e corrido ne, tipo assim para todos os pais com certeza a gente tenta dar o máximo dá o melhor assim eu tento pra minha filha ali nosso tento dá o melhor pra ela assim.

DATA: 22/07/2022 ( ) DOCENTE ( ) COLABORADOR (x) ESTUDANTE

**DADOS DE IDENTIFICAÇÃO**

CÓDIGO DE IDENTIFICAÇÃO: P10

IDADE: 34 anos.

ÁREA DE ATUAÇÃO: Técnico de Enfermagem

NÚMERO DE FILHOS: 2 IDADE DOS FILHOS: 4 anos, 7meses.

QUANTOS VÍNCULOS EMPREGATÍCIOS POSSUI ALÉM DA ATIVIDADE NA UNIVERSIDADE? 2

REALIZOU CURSOS DE PATERNIDADE ANTES DO NASCIMENTO DE SEU FILHO? Não

FREQUENTA HÁ QUANTO TEMPO O ÂMBITO UNIVERSITÁRIO? 3 anos.

**QUESTÕES DA PESQUISA**

1) Explique com suas palavras o que significa ser pai para você.
   Experiência única, dá muito trabalho mas e gratificante.
2) Como você cuida de seu filho com menos de 2 anos?
   Pouco tempo com ele, a noite, mas gente brinca tenta interagir com ele.
3) Na sua opinião, quais ações que realiza podem promover o desenvolvimento infantil de seu filho menor de 02 anos?
   Estimular a falar, estimular a gatinhar.
4) Quais dificuldades você enfrenta no cuidado com o seu filho com menos de 2 anos?
   Tempo né, por ter dois vínculos e o tempo.
5) Para você, qual é a importância da sua participação no cuidado do seu filho menor de 2 anos?
   A presença de pai né.
6) Você acredita que consegue responder todas as necessidades de seu filho?
   Não porque falta tempo, falta tempo.

DATA: 22/07/2022 ( ) DOCENTE ( ) COLABORADOR (x) ESTUDANTE

DADOS DE IDENTIFICAÇÃO

CÓDIGO DE IDENTIFICAÇÃO: P11

IDADE: 38 anos

ÁREA DE ATUAÇÃO: Enfermeiro

NÚMERO DE FILHOS: 1 IDADE DOS FILHOS: 1ano e 6 meses.

QUANTOS VÍNCULOS EMPREGATÍCIOS POSSUI ALÉM DA ATIVIDADE NA UNIVERSIDADE? 2

REALIZOU CURSOS DE PATERNIDADE ANTES DO NASCIMENTO DE SEU FILHO? Não

FREQUENTA HÁ QUANTO TEMPO O ÂMBITO UNIVERSITÁRIO? Uns 4, 5 anos.

QUESTÕES DA PESQUISA

1) Explique com suas palavras o que significa ser pai para você.

   Ser pai bom, ser pai e ser exemplo pra uma pessoa né e você tomar cuidado com tudo que você faz porque desde um simples palavrão uma mania de você deixar um copo largado na pia alguma coisa assim você acaba sendo espelho ne percebo isso com minha filha ela se espelha muito nas coisas que eu faço então várias coisas tanto eu quanto a mãe dela então a gente percebe que ser pai acho que mais o exemplo prático disso e você ser espelho pra alguém você ser modelo para alguém.

2) Como você cuida de seu filho com menos de 2 anos?

   E bem complicado, eu e a mãe dela, somos da área da saúde então devido a nossa rotina de trabalho, fica várias horas fora de casa, a gente tem um pouco contato com ela, então, pelo período da manhã ela fica na escolinha né, à tarde no finalzinho da tarde a minha sogra apanha ela fica com ela, e no final da noite que a gente vai ter contato com a nossa filha.

3) Na sua opinião, quais ações que realiza podem promover o desenvolvimento infantil de seu filho menor de 02 anos?

Hum, então ela frequenta escola particular e a gente teve algumas orientações a respeito disso, introduzir músicas clássicas, jogos lúdicos e conversa por mais que a gente não consiga entender muito o que ela fala devido à idade, e pintura desenho então são formas que a escola nos passou pra tentar ajudar no desenvolvimento dela, principalmente esses joguinhos de montar, joguinhos de cor de encaixe esses jogos lúdicos até pra desenvolver, toda essa área ligada a artes.

4) Quais dificuldades você enfrenta no cuidado com o seu filho com menos de 2 anos?

   Hum, acho que falta de tempo na verdade, acho que e um problema maior que a gente tem e a falta de tempo, como a gente não consegue passar muito tempo com ela, conversar com ela, instruir né então fica difícil nesse sentido, vamos ver se logo em breve a gente consegue passar mais tempo com ela, mas a falta de tempo, que realmente assim a gente não consegue as vezes corrigir talvez manias que ela está tendo agora ne, infelizmente como eu falei, a gente precisa trabalhar pra poder dar sustento pra ela então não tem outra forma de fazer isso.

5) Para você, qual é a importância da sua participação no cuidado do seu filho menor de 2 anos?

   Acho bem importante, mesmo que ela esteja em escola particular a gente percebe que a escola tem uma função educacional, simplesmente passar educação, educação de uma maneira formal, acho que o pai e mãe acabam repassando essa questão de educação social essa maneira de interagir com as pessoas por exemplo ela acostumada a pedir benção pro pai pra mãe pro tio pra tia pro vô e pra vó, então assim o por mais que ela fique bastante tempo na escola, acho que a função da escola e realmente dar a educação formal e a família educação social, então acho que não compete a escola dar educação ensinar boas maneiras pras crianças, realmente não concordo com isso, por mais que ela fique bastante tempo na escola, mas isso e competência realmente do pai da mãe da família no caso.

6) Você acredita que consegue responder todas as necessidades de seu filho?

Não, não nem eu e nem a mãe dela, a gente e bem consciente a respeito disso e também a gente não tenta comprar ela com dinheiro e nem nada, a gente tentou durante um tempo e percebeu que na verdade ela quer carinho atenção e ficar mais tempo com o pai e a mãe, então a gente se dedica a ficar bastante tempo com ela, infelizmente, eu e a mãe dela na verdade a gente percebe que a gente não consegue dar essa atenção que ela precisa, a gente percebe que ela e um pouquinho carente de atenção, então sempre que a gente ta próximo ela quer ficar muito próximo no colo entendeu, ela não e manhosa nem dengosa mas ela a gente percebe que ela sente essa carência e afeto, então o tempo que a gente tem livre folga, feriado, final de semana, noite a gente acaba passando um tempinho com ela, leva ela num parque, passa um tempo assim dedicado a ela mesmo, não tenta comprar subornar ela com dinheiro nem brinquedo caro porque, a gente percebe que pra ela não tem muita diferença assim, então realmente e mais tempo mesmo, dedicar um tempo ao invés de cobrir a criança de brinquedo passar meia hora sentado num tapete, brincando com ela pintando, fazendo maquiagem na mãe, tentando pintar a unha do pai, dessa forma.

DATA: 22/07/2022 ( ) DOCENTE ( ) COLABORADOR (x) ESTUDANTE

DADOS DE IDENTIFICAÇÃO

CÓDIGO DE IDENTIFICAÇÃO: P12

IDADE: 45 anos

ÁREA DE ATUAÇÃO: Enfermeiro

NÚMERO DE FILHOS: 2 filhos IDADE DOS FILHOS: 2 anos, 6 anos

QUANTOS VÍNCULOS EMPREGATÍCIOS POSSUI ALÉM DA ATIVIDADE NA UNIVERSIDADE? 2 vínculos.

REALIZOU CURSOS DE PATERNIDADE ANTES DO NASCIMENTO DE SEU FILHO? Realizei só no primeiro no segundo não.

FREQUENTA HÁ QUANTO TEMPO O ÂMBITO UNIVERSITÁRIO? 15 anos.

QUESTÕES DA PESQUISA

1) Explique com suas palavras o que significa ser pai para você.

   Ser pai é antes de qualquer coisa uma felicidade muito grande uma chance que a gente tem de ser mais feliz ne um comparativo do que era antes sem os filhos e uma perspectiva muito boa de poder passar valores ajudar na formação de opinião desses seres humanos que estão sob nossa responsabilidade.

2) Como você cuida de seu filho com menos de 2 anos?

   Olhe eu tento cuidar dentro daquilo que eu acredito e sei da melhor forma possível né cuidados com a saúde eu me preocupo muito com a questão da alimentação mais também o cuidado afetivo de sempre estar demostrando carinho, amor, desde pequeno também impondo alguns limites já pensando na questão da educação desse meu filho.

3) Na sua opinião, quais ações que realiza podem promover o desenvolvimento infantil de seu filho menor de 02 anos?

   Acredito que as ações, tudo que a gente faz quando está próximo da criança ele ta percebendo e isso influencia diretamente no desenvolvimento dele, tanto na parte física e de saúde tendo um bom

comportamento alimentar praticas esportiva eles sempre estão de olho e querem fazer igual querem jogar futebol andar de bicicleta tudo que eles veem que o pai está fazendo, também na questão sentimental mesmo na questão educação de respeito e o convívio com a minha esposa, na forma que ele nos vê que ele enxerga o meu tratamento com ela o tratamento com os mais idosos por exemplo tratamento com os meus pais com os meus sogros são detalhes assim que no dia a dia ele ta percebendo isso influencia com certeza ne no crescimento na formação dele então eu acredito que o comportamento da gente como um todo né tudo reflete tudo eles estão percebendo.

4) Quais dificuldades você enfrenta no cuidado com o seu filho com menos de 2 anos?

Dificuldades de falta de tempo muitas vezes para estar junto pra ta acompanhando, cansaço da gente pelo fato de trabalhar bastante então eu acho que essa e a maior dificuldade as demais coisas assim são desafios, a parte financeira também e uma dificuldade grande que eles demandam um gasto financeiro alto, com escola, muitas vezes ficam doentes, medicação e também lembrei de uma coisa que e uma dificuldade que tem e quando eles ficam doentes, que você tem que ficar com eles em casa, ai você fica impedido de ir trabalhar muitas vezes então são desafios que a gente encontra.

5) Para você, qual é a importância da sua participação no cuidado do seu filho menor de 2 anos?

Eu acho que a importância e muito grande tanto pra ele quanto pra min, pra ele porque eu estou contribuindo na formação do caráter dele, dando bons exemplos e eu ganho com isso também porque muitas vezes a gente se corrige pra ter uma postura mais adequada frente ao seu filho muitas vezes a gente não e tudo aquilo que o filho ta vendo, muitas vezes a gente tem hábitos que não são saudáveis, comportamento que não são saudáveis mas que a partir do momento que você tem um filho e assumi essa responsabilidade e quer fazer o melhor pra ele, muitas vezes você não e você mesmo, assim de uma forma figurada vamos dizer, você acaba sendo outra pessoa tentando ser uma pessoa melhor para que ele te veja como um bom exemplo e querendo ou não isso faz com que a

gente no final das contas tenha novos hábitos mais saudáveis postura mais corretas que ficam pra gente.

6) Você acredita que consegue responder todas as necessidades de seu filho?

   Eu acho que não, eu acho que tem muita, algumas coisas a gente não tem o conhecimento não tem o preparo e difícil mensurar assim palpar, fazer algo palpável de falar mas o próprio crescimento desenvolvimento da criança muitas vezes tem coisas que passam desapercebidas, eu acho que seria impossível dar conta de tudo cem por cento, acredito que não.

DATA:23/07/2022 (X) DOCENTE ( ) COLABORADOR ( ) ESTUDANTE

**DADOS DE IDENTIFICAÇÃO**

CÓDIGO DE IDENTIFICAÇÃO: P13

IDADE: 45 anos.

ÁREA DE ATUAÇÃO: Docente

NÚMERO DE FILHOS: 3 IDADE DOS FILHOS: 22 anos, 10 anos e 1anos e 3meses.

QUANTOS VÍNCULOS EMPREGATÍCIOS POSSUI ALÉM DA ATIVIDADE NA UNIVERSIDADE? 3

REALIZOU CURSOS DE PATERNIDADE ANTES DO NASCIMENTO DE SEU FILHO? Não.

FREQUENTA HÁ QUANTO TEMPO O ÂMBITO UNIVERSITÁRIO? 5 anos.

**QUESTÕES DA PESQUISA**

1) Explique com suas palavras o que significa ser pai para você.
   Ser pai para min é como eu posso explicar com minhas palavras eu acho muito gratificante eu gosto tento me envolver o máximo com a criação das filhas porque são todas meninas, mas como eu trabalho muito né, somente nas minhas folgas que eu consigo ficar junto com elas.
2) Como você cuida de seu filho com menos de 2 anos?
   Como eu expliquei na pergunta anterior como eu tenho pouco tempo mas no momento que eu to em casa normalmente a gente faz atividades, atividades assistir vídeos educativos a gente sai, a gente vai pros parques, levo ela no âmbito que ela mais gosta saio eu tento sair com ela e levar ela.
3) Na sua opinião, quais ações que realiza podem promover o desenvolvimento infantil de seu filho menor de 02 anos?
   Então, o que eu gosto muito de fazer, como eu falei, gosto de passar pra ela esses vídeos educativos, tudo que ela possa ter um crescimento, como eu não tive na minha vida no caso, eu precisava de alguma coisa

assim também e não tive esse suporte então dou esse suporte pra ela pode evoluir bem.

4) Quais dificuldades você enfrenta no cuidado com o seu filho com menos de 2 anos?

   Dificuldade que eu me sinto muito ausente, essa minha ausência derrepente pode causar alguma coisa no crescimento, tudo nela.

5) Para você, qual é a importância da sua participação no cuidado do seu filho menor de 2 anos?

   Eu tenho que, eu penso assim o pai tem que estar sempre presente, eu tento ficar presente no maior tempo possível que eu tenho nas minhas folgas, então o que eu tenho que passar pra ela tudo aquilo o que eu poderia passar se eu tivesse presente mais dias com ela eu tento passar nesse período que é 12 ou 24 horas que eu to com ela, referente aos meus plantões.

6) Você acredita que consegue responder todas as necessidades de seu filho?

   Não, porque pra eu fazer isso eu deveria estar mais tempo com ela, infelizmente conforme meus plantões eu não consigo.

DATA: 02/08/2022 (x) DOCENTE ( ) COLABORADOR ( ) ESTUDANTE

**DADOS DE IDENTIFICAÇÃO**

CÓDIGO DE IDENTIFICAÇÃO: P14

IDADE: 48

ÁREA DE ATUAÇÃO: Docente da área de geografia

NÚMERO DE FILHOS: 03 IDADE DOS FILHOS: 17, 13, 03

QUANTOS VÍNCULOS EMPREGATÍCIOS POSSUI ALÉM DA ATIVIDADE NA UNIVERSIDADE? (outras universidades ou empregos)
não há outro vínculo

REALIZOU CURSOS DE PATERNIDADE ANTES DO NASCIMENTO DE SEU FILHO?
Cursos não. Assisti palestras, li alguns livros e ouvi conselho de pessoas próximas.

FREQUENTA HÁ QUANTO TEMPO O ÂMBITO UNIVERSITÁRIO?
20 anos

**QUESTÕES DA PESQUISA**

1) Explique com suas palavras o que significa ser pai para você.
   Pai é aquele que ama, cuida, protege, sustenta, educa, repreende, mostra o caminho certo, auxilia e orienta nas descobertas da vida.

2) Como você cuida de seu filho com menos de 2 anos?
   Cercando ele de cuidados, afeto, carinho e muita atenção. Alimentando-o, dando banho, colocando para dormir. Ensinando primeiros passos e primeiras palavras.

3) Na sua opinião, quais ações que realiza podem promover o desenvolvimento infantil de seu filho menor de 02 anos?
   Acompanhar, ensinar a alimentar, a falar e a andar. Contando histórias, lendo livros para ele, sorrindo junto, buscando interações diversas.

4) Quais dificuldades você enfrenta no cuidado com o seu filho com menos de 2 anos?

   Trocar as fraldas. Quando fica doente exige mais atenção.

5) Para você, qual é a importância da sua participação no cuidado do seu filho menor de 2 anos?

   Participo bastante e considero muito importante a presença do pai, diariamente. A minha presença traz segurança e vínculo afetivo.

6) Você acredita que consegue responder todas as necessidades de seu filho? (solicitar que desenvolva a ideia)

   Todas as necessidades não. Consigo suprir as básicas (a minha presença, alimentos, roupas, conforto, moradia, um pouco de lazer) e oferecer algo a mais dentro das possibilidades.

DATA: 03/08/2022 ( ) DOCENTE ( ) COLABORADOR (X) ESTUDANTE

**DADOS DE IDENTIFICAÇÃO**

CÓDIGO DE IDENTIFICAÇÃO: P15

IDADE: 32

ÁREA DE ATUAÇÃO: Condutor de Ambulância

NÚMERO DE FILHOS: 1 IDADE DOS FILHOS: 2

QUANTOS VÍNCULOS EMPREGATÍCIOS POSSUI ALÉM DA ATIVIDADE NA UNIVERSIDADE? (outras universidades ou empregos)

REALIZOU CURSOS DE PATERNIDADE ANTES DO NASCIMENTO DE SEU FILHO?

Não

FREQUENTA HÁ QUANTO TEMPO O ÂMBITO UNIVERSITÁRIO?

**QUESTÕES DA PESQUISA**

1) Explique com suas palavras o que significa ser pai para você.
   Ter um filho é como se o mundo começasse novamente, mudamos completamente a maneira de ver as coisas, entendemos os sentimentos de nossos pais, só queremos melhorar cada dias e dar o melhor pra a criança.

2) Como você cuida de seu filho com menos de 2 anos?
   Busco cuidar da maneira mais saudável possível, sempre presente no pediatra dela, mas como é minha primeira e única filha, muitas vezes me pego no desespero, não sei o que fazer, então recorro as dicas dos meus pais e dos pais da minha esposa, já que passaram por isso algumas vezes, assim fico mais tranquilo.

3) Na sua opinião, quais ações que realiza podem promover o desenvolvimento infantil de seu filho menor de 02 anos?

Brinquedos educativos, lendo livro infantis para minha filha, mostrando as figuras, envolvendo-a com mais crianças, afim de ter uma boa interação social.

Levo ela para conhecer a natureza, ver e interagir com os animais.

4) Quais dificuldades você enfrenta no cuidado com o seu filho com menos de 2 anos?

   A maior dificuldade é em entender o que ela quer ou se está com alguma dor, mas insistimos até conseguir entender os sinais que ela nos passa, então recorremos de acordo com a necessidade.

5) Para você, qual é a importância da sua participação no cuidado do seu filho menor de 2 anos?

   Acredito que a maior importância é estar presente, não importa a situação, se você sebe ou não o que fazer, mas estar presente demostrando amor e mostrando pra ela que você está ali com ela.

6) Você acredita que consegue responder todas as necessidades de seu filho? (solicitar que desenvolva a ideia)

   Eu tento responder, mas acredito que ainda falta muito não sei se são realmente necessidades ou somente coisas que quero ser ou fazer melhor.

   Mas no geral, acredito que o necessário para desenvolvimento, dar amor, atender na saúde dela, eu estou respondendo.
