# Supplementary material for: Men’s perception of paternal parenthood and the promotion of child development
Source: Rev Bras Enferm. 2024 Jul 29;77(3):e20230514. doi: 10.1590/0034-7167-2023-0514 (PMC11290730; doi:10.1590/0034-7167-2023-0514)
Supplement: Translation

# Translation: A percepção dos homens sobre a parentalidade paterna e a promoção do desenvolvimento infantil

**DOI:** 10.1590/0034-7167-2023-0514pt

**Authors:** Gustavo Selenko de Aquino, Sarah de Almeida Rocha Moura, Ailton de Lima, Samara Macedo Cordeiro, Jéssica Batistela Vicente, Verônica de Azevedo Mazza

**Affiliations:** I Complexo Hospital de Clínicas e Maternidade Victor Ferreira do Amaral - filiado a EBSERH, Unidade da Mulher. Curitiba, Paraná, Brasil; II Irmandade da Santa Casa de Misericórdia. Curitiba, Paraná, Brasil; III Prefeitura Municipal de Curitiba. Curitiba, Paraná, Brasil; IV Universidade Estadual de Campinas. Campinas, São Paulo, Brasil; V Universidade Federal do Paraná. Curitiba, Paraná, Brasil

**Keywords:** Poder Familiar, Paternidade, Cuidado da Criança, Desenvolvimento Infantil, Lactente

## Abstract

**Objetivos:**

compreender a percepção dos homens em relação à parentalidade paterna exercida no cuidado aos lactentes para a promoção do desenvolvimento infantil.

**Métodos:**

estudo qualitativo, de abordagem exploratória, realizado com discentes, docentes e técnicos administrativos que são pais de lactentes com até 6 meses de vida de uma instituição de ensino superior, sendo excluídos pais de famílias mononucleares. A coleta de dados ocorreu por meio de entrevista semiestruturada e foi analisada à luz da análise categorial temática.

**Resultados:**

participaram da pesquisa 15 homens. A partir da análise, emergiram duas categorias empíricas: “Percepção sobre ser pai: desafios e novidades” e “Promoção do desenvolvimento infantil: ações realizadas pelos pais”. Os pais revelaram sentir-se despreparados, a necessidade de apoio emocional e reconhecem as atividades promovidas para o desenvolvimento de seus filhos.

**Considerações Finais:**

destaca-se a relevância da figura paterna para o desenvolvimento infantil e a necessidade de políticas públicas de incentivo à parentalidade paterna.

## INTRODUÇÃO

O desenvolvimento infantil é um processo ativo e único de cada criança, evidenciado pela continuidade nas mudanças nas habilidades motoras, cognitivas, socioemocionais e de linguagem, com ganhos progressivos nas habilidades, das mais simples às mais complexas, nas funções da vida diária e no exercício de seu papel social^(1)^. A primeira infância (0 aos 6 anos de vida)^(2)^ tem sido reconhecida como momento estratégico para o desenvolvimento humano, uma vez que, nesse período, o cérebro da criança encontra-se em intensa expansão e tem alta plasticidade^(3)^. Esse é o período de maior risco quando a criança está exposta a situações adversas ao seu desenvolvimento, mas também é a fase com melhores resultados de ações que promovam habilidades motoras, cognitivas, de linguagem e socioemocionais^(4-5)^.

O desenvolvimento infantil é influenciado pelo ambiente e contexto em que a criança está inserida. Assim, a ausência da figura materna ou paterna pode acarretar consequências negativas para o desenvolvimento global da criança ao longo da vida^(6-7)^. Entre as consequências, observa-se o atraso e dificuldades na fala, na interação social, no raciocínio lógico, na adesão à realização de atividades físicas, dificuldade de concentração e maturação emocional^(1)^.

No que diz respeito à figura paterna, nota-se que os homens sustentam e facilitam a relação entre mãe e filho, tanto material quanto emocionalmente. Materialmente, os homens possibilitam aspectos financeiros e práticos, como garantia das necessidades básicas e compartilhamento com a mãe de algumas responsabilidades, como cuidado com os outros filhos e cuidados com a casa. Emocionalmente, os pais favorecem o desenvolvimento de vínculo emocional entre a mãe e o bebê e participam do cuidado à criança com suporte emocional ao binômio mãe-bebê^(8)^.

Em uma revisão sistemática que buscou analisar as intervenções parentais para a promoção ao desenvolvimento infantil para menores de 3 anos, observou-se que a maioria das intervenções (93%) focou exclusivamente nas mães, enquanto 7% envolveram os pais em certa medida. Compreende-se que mesmo com estudos incluídos em 33 países diferentes, sendo eles majoritariamente de alta renda, o pai ainda é pouco envolvido em ações para a promoção do desenvolvimento infantil^(9)^.

Em um levantamento comparativo realizado entre cinco países (Brasil, Espanha, Austrália, Estados Unidos e Portugal), observou-se que os programas de promoção da parentalidade positiva estão inseridos principalmente no contexto da violência doméstica perpetrada por homens. Esta compreensão permite ao profissional de saúde intervenções socioemocionais mais efetivas e direcionadas no cuidado às famílias, sendo essa uma estratégia fundamental no campo das políticas públicas^(10)^.

No Brasil, a Política Nacional de Atenção à Saúde do Homem, em suas diretrizes, busca promover e discutir questões relacionadas à parentalidade paterna responsável e inclusão do homem nos cuidados com os filhos, por meio da sensibilização de gestores, profissionais de saúde e da população em geral sobre os benefícios ao desenvolvimento infantil, a partir do envolvimento ativo dos homens^(11)^.

O Marco Legal da Primeira Infância, lei federal brasileira que estabeleceu princípios e diretrizes para a formulação de políticas públicas e programas para crianças até 6 anos de idade, ressalta a importância da presença de um cuidador responsivo e que atenda às necessidades das crianças. A Política Nacional de Atenção Integral à Saúde da Criança e do Adolescente (PNAISC) brasileira também reforça a importância da presença dos pais para o desenvolvimento integral da criança^(2,12)^.

Mundialmente, o desenvolvimento infantil também tem sido uma prioridade das autoridades e a estratégia *Nurturing Care*, lançada pela Organização Mundial de Saúde (OMS) em 2018 como um conjunto de ações globais direcionadas aos cuidados responsivos às crianças, aponta o desenvolvimento infantil (DI) como um dos pilares para atingir os Objetivos de Desenvolvimento Sustentável (ODS) até 2030^(13)^.

Entre os benefícios da participação paterna para o desenvolvimento infantil estão a segurança, o fortalecimento do vínculo pai-filho/familiar, a formação da personalidade, a maturação do caráter ético-moral e um melhor desenvolvimento socioemocional^(14)^.

Entre as ações que promovem a parentalidade positiva e fortalecem as competências parentais estão o desenvolvimento, pelos profissionais na Atenção Primária à Saúde, de consultas de pré-natal, visitas domiciliares, consultas de puericultura e grupos educativos, entre outros, e estas devem ser promovidas em todos os espaços de cuidados^(15)^.

Para que se possa pensar em ações de cuidado que fortaleçam o exercício da parentalidade desenvolvida por homens, é relevante compreender suas percepções sobre esse papel. Nota-se que os estudos atuais sobre parentalidade paterna estão voltados para as áreas de pré-natal e para a compreensão do papel do homem nos cuidados antes do nascimento da criança, sendo necessários estudos que possam compreender a atuação dos homens na promoção do desenvolvimento infantil^(16)^.

## OBJETIVOS

Compreender a percepção dos homens em relação à parentalidade paterna exercida no cuidado aos lactentes para a promoção do desenvolvimento infantil.

## MÉTODOS

### Aspectos éticos

O estudo foi conduzido de acordo com as diretrizes éticas nacionais e internacionais e aprovado pelo Comitê de Ética da Universidade Cesumar. O Consentimento Livre e Esclarecido foi obtido de todos os participantes do estudo. Para preservar o anonimato, o nome dos participantes foi substituído pela abreviação “P” para Pai entrevistado, seguido de números de um a quinze, de acordo com a ordem das entrevistas.

### Tipo de estudo

Trata-se de um estudo de abordagem qualitativa exploratória^(17)^. Foi utilizado o instrumento *Consolidated Criteria for Reporting Qualitative Research* (COREQ)^(18)^ para orientação da metodologia.

### Seleção dos participantes e cenário de pesquisa

Os participantes foram selecionados por conveniência e convidados a participar do estudo durante o intervalo das aulas em uma universidade do sul do Brasil, após explicação do objetivo do estudo. Participaram 15 pais, entre eles docentes, discentes e técnicos administrativos na instituição. O critério de inclusão foi ter filhos com idade entre 6 meses e 2 anos de vida. Foram excluídos pais de família mononuclear, para que se pudesse compreender a percepção de cuidado pelos homens na execução da atividade parental, onde a mãe também está presente. Não houve recusa de participantes.

### Coleta de dados e organização dos dados

A coleta de dados ocorreu nos meses de fevereiro a julho de 2022, por meio de entrevista semiestruturada, audiogravada de forma individual, presencial e em espaço reservado. As perguntas norteadoras da pesquisa foram: “Explique com suas palavras o que significa ser pai para você”, “Como você cuida de seu filho com menos de 2 anos?”, “Na sua opinião, quais ações que você realiza podem promover o desenvolvimento infantil de seu filho menor de 2 anos?”, “Quais dificuldades você enfrenta no cuidado com o seu filho com menos de 2 anos?”, “Para você, qual é a importância da sua participação no cuidado do seu filho menor de 2 anos?” e “Você acredita que consegue responder a todas as necessidades de seu filho?”.

Além das perguntas, foi realizada coleta de dados sociodemográficos para caracterização dos participantes. As entrevistas tiveram duração média de 10 minutos e foi realizado apenas um encontro com cada participante.

O roteiro de coleta de dados foi testado previamente, e esta entrevista não foi incluída nos resultados apresentados. As entrevistas foram conduzidas por dois acadêmicos treinados pelo pesquisador principal, sendo um do sexo feminino e outro masculino do último ano do curso de Enfermagem, que não possuíam relação direta com os entrevistados. O convite para participar da entrevista foi realizado antes do início da aula ou no intervalo. A coleta de dados foi encerrada quando os objetivos haviam sido alcançados e as perguntas de pesquisa respondidas.

### Análise de Dados

As entrevistas foram transcritas na íntegra e analisadas por meio da técnica de Análise de Conteúdo, na modalidade temática^(19)^. A análise foi realizada em três passos: a pré-análise, que consiste na organização das ideias por meio da leitura flutuante do material, estabelecimento de hipóteses e objetivos, e elaboração de indicadores que fundamentam a interpretação final; a exploração do material, em que foi realizada a codificação dos dados brutos do texto; e a categorização dos dados, em que foi realizado o tratamento dos dados, inferência e interpretação, por meio de isolamento/diferenciação dos elementos em planilhas do *Excel* ^®^, com posterior reagrupamento conforme as semelhanças entre si.

Após a conclusão da pesquisa, os participantes foram convidados a participar da mostra de trabalhos de conclusão do curso de Enfermagem, na qual foi realizada a apresentação dos resultados da pesquisa.

## RESULTADOS

Participaram da pesquisa 15 homens, com idades entre 25 e 40 anos, sendo 9 discentes, 4 docentes e 2 técnicos administrativos do ensino superior. As profissões mencionadas pelos discentes e técnicos administrativos, além do vínculo com a instituição de coleta de dados, foram: Condutor de Ambulância (3), Enfermeiro (3), Técnico de Enfermagem (2), Rádio Operador do Serviço de Atendimento Móvel de Urgência - SAMU (1), Dentista (1) e Analista de Sistemas (1). Quanto ao número total de filhos, 3 pais tinham três filhos, 6 tinham dois filhos e 6 tinham um filho. Com relação à idade dos filhos dos participantes, variaram entre 7 meses e 22 anos. Quando questionados sobre a participação em cursos de promoção da parentalidade paterna, somente 2 entrevistados relataram terem participado.

Após a análise das falas, emergiram duas categorias: “Percepção sobre ser pai: desafios e novidades” e “Promoção do desenvolvimento infantil: ações realizadas pelos pais”.

### Percepção sobre ser pai: desafios e novidades

Muitos pais revelam sentir-se despreparados para enfrentar os desafios da parentalidade, enfrentando alguns obstáculos. Apesar das dificuldades, eles evidenciam a importância de serem exemplos éticos e morais para seus filhos, ilustrando responsabilidade e dedicação ao exercício da parentalidade. Os participantes descrevem sua experiência em ser pai como desafiadora, nova e gratificante ao cuidar e proteger o filho:

*Ser pai para mim é um desafio principalmente na atualidade, principalmente durante e pós pandemia. Ser pai é a parte mais difícil, é educar os filhos. Ao mesmo tempo, tentar protegê-los e blindá-los de algumas coisas que esse mundo tem é um dos maiores desafios. É a parte de educação, é a que mais pesa.* (P8)

*Experiência única, dá muito trabalho mas é gratificante.* (P10)

*Ser pai para mim é muito gratificante, eu gosto e tento me envolver ao máximo com a criação das minhas filhas, porque são todas meninas.* (P13)

*Pai é aquele que ama, cuida, protege, sustenta, educa, repreende, mostra o caminho certo, auxilia e orienta nas descobertas da vida.* (P14)

A falta de preparo para exercer a parentalidade paterna foi relatada como a dificuldade de atender às necessidades dos filhos, a pressão social da responsabilidade da parentalidade e a influência em suas vidas:

*Eu tento, se conseguir sanar todas* [as necessidades] *aí já é uma outra parte. Porque sempre vai ter alguma dificuldade, mas tento da melhor forma possível.* (P2)

*Ser pai foi uma coisa que veio assim, em um momento que não esperava. Para mim, achei de início que não iria ser capaz de cuidar de um ser que nos transforma. Pois querendo ou não, muda tudo. Não tenho como explicar, é uma coisa única, um conjunto de sentimentos que a gente desenvolve.* (P5)

[...] *eu acho que tem algumas coisas que não tenho conhecimento e nem preparo. É difícil mensurar, fazer algo palpável, de falar, mas o próprio crescimento e desenvolvimento da criança muitas vezes tem coisas que passam despercebidas. Eu acho que seria impossível dar conta de tudo cem por cento.* (P12)

*Muitas vezes me pego no desespero, não sei o que fazer.* (P15)

Além da falta de preparo, a falta de tempo para exercer a parentalidade paterna foi identificada pelos pais, visto que não conseguem estar presentes devido a compromissos com o trabalho:

*Me falta tempo, por causa dos trabalhos. Eu passo um tempo ausente, então acho que isso deixa uma lacuna.* (P1)

[...] *não consigo atender todas as necessidades dela porque depende do serviço, da faculdade, estou correndo, estudando e tudo. Então acabo não dedicando todo o tempo que eu queria me dedicar a ela. Gostaria de ser um pouco mais presente, um pouco mais ativo mas infelizmente, não consigo responder a altura.* (P5)

*Não consigo porque me falta tempo, devido aos meus dois vínculos. Não consigo suprir tudo, porque eu deveria ter que trabalhar menos e passar mais tempo para que conseguisse desenvolver isso* [cuidado] *melhor.* (P8)

*Eu trabalho muito, somente nas minhas folgas que eu consigo ficar junto delas.* (P13)

Entre as novidades, os participantes também relatam a preocupação em ser exemplo ético e moral para o desenvolvimento de seus filhos:

[...] *ter a responsabilidade de cuidar de uma criança, cuidar de todas as formas, dando afeto, educando, dando bom exemplo, fazendo com que principalmente siga seus bons exemplos para ser alguém na vida. Para ter futuro.* (P1)

[...] *eu acho que a primeira coisa a passar nessa idade, é o que é certo e errado. O que pode o que não pode.* (P2)

[...] *A figura paterna e a figura materna são centrais na vida da criança. Eles fazem uma referência para o resto da vida dela,* [...]. (P3)

*Ser exemplo para uma pessoa é você tomar cuidado com tudo que você faz. Porque desde um simples palavrão, até uma mania de você deixar um copo largado na pia ou alguma coisa assim, você acaba sendo espelho.* (P11)

### Promoção do desenvolvimento infantil: ações realizadas pelos pais

Os participantes relataram sobre a necessidade do apoio emocional, ações de cuidado no cotidiano e ainda referiram atividades promovidas para o desenvolvimento socioemocional de seus filhos. O cuidado próximo e afetuoso foi apontado como aquele que oferece apoio emocional e promove o vínculo afetivo por meio da presença paterna e das demonstrações que transmitem à criança, como o carinho e conforto emocional:

[...]*estar presente, sentir que quer minha presença. Tive pais presentes, e com ele não vai ser diferente. Para suprir todas* [necessidades] *eu teria que estar cem por cento com ele. Sempre falta alguma coisa, mas no cenário que convivo consigo suprir o máximo que ele necessita.* (P7)

*Participo bastante e considero muito importante a presença do pai diariamente. A minha presença traz segurança e vínculo afetivo.* (P14)

*Acredito que a maior importância é estar presente, não importa a situação, se você sabe ou não o que fazer, mas estar presente demonstrando amor e mostrando para ela que você está ali com ela.* (P15)

Ainda foi referido pelos pais o cuidado em busca da saúde da criança, auxílio nas atividades cotidianas como alimentação, banho, consultas médicas e estímulo ao tempo de lazer:

*Eu cuido, dou banho, alimento, educo, ensino, brinco com ele, e quando preciso corrigir, faço as correções* (P1)

*Nos momentos que temos para ficar juntos, como a nossa vida e meio corrida, tento aproveitar da melhor maneira possível. Dou carinho, atenção, tento estar sempre presente com ela, eu mesmo, troco e dou alimentação, dou mama.* (P2)

*Eu cuido dela. Nos dias em que eu estou em casa nós brincamos, nos divertimos. Ajudo na alimentação nos cuidados com alguns afazeres. Procuro trazer para mim o banho, alimentação e tento gastar esse tempo que eu tenho livre com elas.* (P7)

*Busco cuidar da maneira mais saudável possível, sempre presente no pediatra dela, mas como é minha primeira e única filha* […] *então recorro às dicas dos meus pais e dos pais da minha esposa. Já que passaram por isso algumas vezes, assim fico mais tranquilo.* (P15)

Nota-se ainda que os pais procuram manter atividades que estimulem a criança no desenvolvimento do raciocínio e da linguagem, repetindo frases, palavras e/ou perguntas, além de adotar atitudes com o intuito de promover a independência da criança. Já os estímulos cognitivos e socioemocionais utilizados pelos pais incluem a pintura, o desenho, os jogos de memorização, vídeos na internet, a leitura de livros, o uso da música, a interação com os animais e o contato com a natureza:

*Brincar com ele, não aderir tanto à modernidade de hoje.* (P4)

[...] *tento ir ensinando o básico, algumas letras, números, algumas coisas para ela tentar ir desenvolvendo por meio da brincadeira.* (P5)

*Interação, o brincar, e principalmente o afeto.* (P6)

[...] *olha eu acho que o que desenvolve é a parte de brincadeiras lúdicas. As brincadeiras que temos em casa, musicalização e desenho* (P8)

[...] *brincadeiras, músicas. Agora com a internet tudo fica mais fácil, aliás às vezes é uma musiquinha, se está chorando já coloca ali, já desenvolve ela. É um desenhinho, apesar de que ela não é muito de desenho. Ela gosta mais da música e o Miguel gosta mais dos videozinhos. Ele já tem os desenhos que gosta.* (P9)

*Estimular a falar, estimular a engatinhar.* (P10)

*Ela frequenta a escola particular e tivemos algumas orientações a respeito disso. Introduzir músicas clássicas, jogos lúdicos e conversar. Por mais que não consiga entender muito o que ela fala devido à idade. A pintura, o desenho, são formas que a escola nos passou para ajudar no desenvolvimento dela, principalmente esses joguinhos de montar. Joguinhos de cor de encaixe esses jogos lúdicos até para desenvolver, toda essa área ligada a artes.* (P11)

## DISCUSSÃO

Os participantes apresentam idade, ocupação e número de filhos diversificados, proporcionando uma visão abrangente da parentalidade paterna em diferentes estágios da vida e contextos profissionais. Notavelmente, a presença significativa de profissionais de saúde na pesquisa pode influenciar sua percepção sobre o cuidado com lactentes devido à sua proximidade e compreensão dos cuidados em saúde. A preparação formal para a parentalidade é limitada, com apenas dois pais tendo participado de cursos de promoção da parentalidade paterna, sugerindo que a maioria dos pais pode não estar adequadamente preparada para o cuidado dos filhos. Além disso, a maioria dos homens mantém dois vínculos empregatícios, indicando jornadas de trabalho extensas e múltiplas responsabilidades, o que afeta o tempo e a energia disponíveis para dedicar aos filhos.

Ainda são escassas as pesquisas que apresentam dados epidemiológicos sobre a relação e participação de homens no cuidado à criança. No último relatório apresentado pela *Helping Dads Care* ^(20)^, sobre os motivos dos homens usufruírem do afastamento de licença paternidade, no ano de 2018, menos de 1/3 dos pais relataram que trabalhavam em empresas que não garantia esse direito. A pesquisa evidenciou que 64% da amostra mostrou-se interessada em estender a licença de 5 para 20 dias, conforme estabelecido na lei Empresa Cidadã. Quando questionados sobre a satisfação com a política de licença paternidade no Brasil, 87% dos homens e 93% das mulheres se dizem satisfeitos. Quando questionados sobre o envolvimento no cuidado dos filhos, 37% dos homens dizem não ter tempo suficiente para realizar ações de cuidados que promovam o desenvolvimento dos filhos e 59% se dizem satisfeitos com o seu envolvimento com seu filho.

Nota-se que são necessárias pesquisas que discutam a política de licença parental de conciliação entre o cuidado com os filhos e o trabalho dos homens, conforme é apresentado pelo Plano Nacional para a Primeira Infância (PNPI) do Brasil^(21)^.

O PNPI do Brasil é um guia político e técnico que orienta a proteção e promoção dos direitos das crianças nos primeiros seis anos de vida, destacando a importância da compreensão social da criança como sujeito de direitos. Suas ações abrangem a integração com os Planos Nacionais de Educação e Saúde, contemplando direitos da criança de 0 a 6 anos, como a convivência familiar, saúde mental, brincar e registro civil, por exemplo. Na última revisão, houve a inclusão do papel do homem no cuidado à família, especialmente no contexto do Pré-Natal do Parceiro na Atenção Primária, visando o envolvimento masculino durante a gestação, além de propostas para cursos de pós-graduação sobre desenvolvimento infantil e parentalidade^(21)^.

A parentalidade paterna é referida pelos pais do presente estudo como uma experiência desafiadora, sendo descrita a adaptação a uma nova fase de vida, com responsabilidades crescentes e as pressões sociais e pessoais associadas ao papel de pai. Apesar dos desafios e novidades, a parentalidade paterna é apresentada também como uma jornada nova e gratificante. A construção histórica entre os papéis de homens e mulheres ocorre de formas diferentes. Enquanto aos homens são direcionadas as responsabilidades financeiras e materiais, no contexto das mulheres são atribuídas as demandas emocionais, sendo elas colaborativas para o desenvolvimento emocional das crianças^(22)^. A ênfase é dada ao papel ativo que os pais têm no cuidado cotidiano, que vai além de prover sustento ou ser uma figura autoritária. O cuidado abrange diferentes aspectos do desenvolvimento e bem-estar da criança, sendo exemplificado pelos cuidados diários como alimentação e presença nas consultas médicas.

Nota-se que apesar das abordagens legislativas brasileiras, ainda é baixo o número de políticas em vigor que incentivam a parentalidade paterna, o que pode influenciar a presença e participação do pai no cuidado da criança nos primeiros anos de vida. As políticas públicas estão em constantes discussões para melhor atender as necessidades da prática paterna. Atualmente, as políticas públicas brasileiras que abordam a promoção da parentalidade paterna são: Licença Paternidade; Lei do acompanhante à parturiente e puérpera; Licença adotante; Estatuto da criança e do adolescente e a Política Nacional de Atenção Integral à Saúde do Homem (PNAISH)^(2)^. No que concerne aos atos legais de promoção da parentalidade paterna, é observada uma lacuna científica que promova um aprofundamento teórico sobre o tema e o desenvolvimento infantil, mesmo existindo o Plano Nacional pela Primeira Infância desde 2010.

Além do cuidado físico e prático, a parentalidade paterna também abrange a dimensão emocional, que envolve a demonstração de carinho, amor, compreensão, entre outras formas de expressão emocional. As políticas públicas e os empregadores são fundamentais para promover um melhor equilíbrio entre carreira e parentalidade, pois as consequências dos estereótipos da masculinidade hegemônica e do trabalhador ideal (priorizando o trabalho em detrimento de outros interesses) podem impactar negativamente no equilíbrio entre o profissional e a parentalidade, impedindo que os homens adotem modelos de parentalidade mais realistas e cuidadosos^(23)^.

O último relatório lançado pela *United Nations International Children’s Emergency Fund* - UNICEF^(24)^ apresentou os dados referentes à situação da licença parental no mundo. Até o ano de 2015, 48% dos países do mundo não possuíam disponibilidade de licença parental paga (incluindo licença maternidade). Ainda neste relatório, 68% dos países de renda alta têm a licença paga para ambos os pais, contra 38% e 47% nos países de renda baixa e média, respectivamente. Cerca de 22% dos países fornecem pelo menos 4 semanas de licença parental, e somente 3% dispõem da transferência da licença para o pai quando a mãe é ausente, sem essa possibilidade existir por escolha do casal.

Nestas condições, é importante ressaltar que para a construção da mudança cultural e promoção das condições adequadas ao desenvolvimento infantil, são necessárias políticas públicas específicas para a parentalidade paterna, bem como iniciativas regionalizadas que proponham melhorias para o acesso ao exercício da parentalidade. Um exemplo disso é o projeto *MenCare*, uma iniciativa global que tem o objetivo de ampliar as discussões sobre a equidade de gênero e promover a inserção dos homens nos cuidados com as crianças, promovendo mudanças sociais^(20)^.

Os participantes do estudo descrevem como ações cotidianas que realizam a alimentação, o banho e brincadeiras, sendo estas essenciais para a prática da parentalidade paterna. Mas há de se considerar que os cuidados à criança ainda estão diretamente ligados à figura materna. Ressalta-se que o envolvimento emocional entre pai e filho é imprescindível para o crescimento e desenvolvimento infantil e que a identidade do sujeito é estabelecida na infância, construindo seus referenciais de moralidade e ética^(25)^.

Compreende-se que o envolvimento paterno está diretamente atrelado ao grau de vínculo exercido junto à criança. Pesquisa aponta que pais que possuem um apego seguro envolvem-se de maneira mais significativa nos cuidados, se comparados aos pais com apego inseguro. Dentre isso, os pais com apego seguro conseguem incentivar os filhos no desenvolvimento de atividades desafiadoras, por meio da realização de cuidados como dar banho e levar à escola^(25)^.

Em revisão sistemática realizada^(6)^, foi constatado que o envolvimento paterno possui influência direta sobre o estímulo verbal, cuidado e interação física com a criança. No que diz respeito ao grau de instrução paterno, pais com formação universitária demonstram maior engajamento em atividades verbais, enquanto a presença de conflito conjugal está correlacionada com uma menor participação em responsabilidades de cuidado e atividades físicas com os filhos.

Devido ao período de construção e maturação biopsicossocial, para que as crianças não apresentem disfunções socioemocionais e intelectuais futuras, é necessário promover boas condições à criança para que haja equilíbrio para o desenvolvimento de autoconfiança. Um indivíduo com maturidade irá desempenhar adequadamente sua autonomia e obter relacionamentos interpessoais de forma positiva^(26)^.

O ambiente deve proporcionar uma experiência embasada em conforto emocional por meio da sensibilidade do pai e/ou cuidador durante a infância, promovendo apego e contribuindo para atender as necessidades da criança. Ressalta-se ainda a necessidade de ajuda na resolução de conflitos, para assim promover o desenvolvimento de habilidades sociais e emocionais, junto à autorregulação definindo a percepção de si mesmo, proporcionando uma presença ativa e de qualidade entre pai e filho^(26)^.

A OMS, para a melhoria deste cenário, desenvolveu uma cartilha para pais, que propõe direcionar as atribuições e ações para desempenhar a parentalidade. Assim, reforça-se a importância da parentalidade paterna para promover ações que vão além do cuidado físico, mas que também englobam o envolvimento emocional, garantindo maior vínculo afetivo e assegurando a promoção do desenvolvimento infantil^(27)^.

A falta de habilidades dos homens, aliada à insegurança sobre sua capacidade de agir com as crianças, sugere uma falta de orientações na Atenção Primária em Saúde^(28)^. Entretanto, observa-se que ainda há pouca procura por parte dos homens para o aprendizado sobre os cuidados com o recém-nascido, reafirmando ainda mais a necessidade de realizar orientações, debates e busca ativa para melhor compreensão da importância do seu preparo e participação ativa^(29)^.

Destaca-se a relevância do papel do enfermeiro ao realizar as consultas de pré-natal e puericultura em convidar o pai para participar das consultas e envolvê-los nos cuidados. É importante que o enfermeiro reconheça a importância do pai para o desenvolvimento do filho e, para tanto, estimule a sua participação desde a consulta de pré-natal, inclusive oferecendo orientações e treinamentos para que ele se sinta ainda mais capaz de conduzir os cuidados junto à família, em casa.

Uma pesquisa que teve como objetivo descrever a competência paterna a partir das orientações fornecidas pela equipe de Enfermagem identificou que os pais de recém-nascidos que receberam instruções pela equipe de Enfermagem se sentiram mais preparados e devidamente instruídos para prestarem os cuidados ao bebê. Essas orientações possibilitaram a compreensão das necessidades a suprir em cada período da sua primeira infância, intervindo e atuando com confiança, proporcionando boas experiências e emoções positivas entre pai e filho, assim garantindo o crescimento e desenvolvimento saudável da criança^(30)^.

Desta forma, a parentalidade paterna é entendida como um processo dinâmico e contínuo, que ocorre por meio do relacionamento do pai com sua família e consigo mesmo, garantindo uma melhor qualidade de vida e vínculos emocionais saudáveis^(31)^. Na presente pesquisa, a falta de tempo para exercer a parentalidade paterna foi mencionada como algo corriqueiro, visto as demandas diárias em conciliar trabalho, estudos e cuidados com os filhos. Equilibrar carreira e parentalidade parece ser um obstáculo real na vida do pai, visto que ainda são escassas políticas públicas que possam amparar este progresso social e cultural, e assim alcançar a equidade de gênero^(32)^.

Os pais reconhecem a importância de atividades que promovam o desenvolvimento integral de seus filhos. Entre as ações referidas estão os estímulos cognitivos e socioemocionais que proporcionam base para o desenvolvimento infantil. Pesquisas têm demonstrado a importância do papel dos pais na promoção do desenvolvimento cognitivo e socioemocional de seus filhos. A presença ativa dos pais na vida cotidiana das crianças aumenta sua oportunidade de aprendizagem e estímulo. Uma das ferramentas que os pais utilizam frequentemente para enriquecer a experiência educativa de seus filhos é a música^(6)^. A exposição à música desde cedo tem o potencial de melhorar a cognição e as habilidades sociais das crianças. Além disso, atividades como desenhar, brincar e interagir com a natureza proporcionam múltiplos benefícios, ajudando a desenvolver a coordenação motora fina e estimulando a criatividade^(33)^.

Os pais, ao falarem e responderem ativamente aos sons emitidos pelos seus filhos, incentivam o desenvolvimento da fala. Além da linguagem, estimular movimentos, como o engatinhar, é essencial para o desenvolvimento motor^(34)^. Interações lúdicas, como ajudar o bebê a se movimentar em diferentes superfícies, podem acelerar esse processo^(35)^.

Os jogos lúdicos não são apenas uma fonte de diversão para a criança, mas também uma poderosa ferramenta de aprendizagem. A brincadeira, especialmente aquela que é imaginativa e não estruturada, promove o desenvolvimento cognitivo, emocional e social das crianças. Além disso, os pais que participam de brincadeiras práticas, como montar blocos ou quebra-cabeças com os filhos, proporcionam oportunidades valiosas para o desenvolvimento do pensamento espacial e das competências de resolução de problemas^(36)^.

Observa-se a necessidade da figura paterna durante o desenvolvimento, e esta pode ser efetivada de diversas formas, a exemplo das brincadeiras, conforme descrito pelos participantes. O brincar é tão importante para uma criança quanto suas necessidades básicas de alimentar-se e dormir, pois é ele que possibilita às crianças explorarem, aprenderem regras sociais e aprofundarem seu desenvolvimento^(37)^. As experiências lúdicas abrangem diferentes faixas etárias e podem incluir jogos, brinquedos e brincadeiras. Essas formas de entretenimento funcionam como uma ferramenta de aprendizagem por meio da qual podem ser expostos sonhos, medos, fantasias e frustrações^(38)^.

A brincadeira desperta diversos processos internos de desenvolvimento na criança e funciona quando a criança interage com as pessoas em ambientes diversos e coopera com os pares. Desta forma, o brincar desempenha um papel importante na aprendizagem e na integração social, pois desperta a emoção e a imaginação, dando forma e densidade à experiência de perceber, sentir e pensar, criando imagens internas que se combinam para representar essa experiência. A imaginação é a raiz de qualquer processo intelectual, seja científico, artístico ou tecnológico. Estas interações sociais precoces, forjadas no contexto da brincadeira, são fundamentais para a formação do indivíduo e, consequentemente, na sua compreensão e prática da moral e ética^(39)^.

A interação com a natureza referida pelos pais como uma das estratégias para a promoção do desenvolvimento infantil tem sido discutida por alguns estudos como uma ação necessária e fundamental para o desenvolvimento infantil. Experiências ecológicas ao ar livre promovem, além do envolvimento emocional entre pais e a exploração de novos locais, contribuem para comportamentos mais sustentáveis. Além de influenciarem uma preocupação ambiental e o comportamento futuro em relação ao meio ambiente, a promoção do brincar na natureza contribui para a conservação da biodiversidade^(40)^.

A construção da parentalidade paterna é um processo construído e vivenciado de forma individual e sofre influência de fatores multissistêmicos. Entre os fatores considerados fundamentais para a promoção da parentalidade paterna, pode-se citar: a saúde individual dos homens, o processo de aprendizagem sobre a parentalidade, aspectos socioeconômicos, apoio social para a promoção de interações positivas, respaldo legislativo e político para fomentar a parentalidade, além da preservação da continuidade cultural. Esses elementos são essenciais para fomentar e promover o envolvimento dos pais na criação dos filhos^(6)^.

### Limitações do estudo

Este estudo apresenta algumas limitações, incluindo a impossibilidade de generalizar os dados como representativos dos pais brasileiros, uma vez que os resultados se referem à experiência individual de homens associados ao meio acadêmico e, em sua maioria, vinculados à área da saúde, o que pode não refletir a diversidade de experiências da parentalidade paterna em variados contextos socioeconômicos e culturais brasileiros. Além disso, a falta de uma exploração mais aprofundada das causas subjacentes à escassez de tempo reportada pelos participantes configura uma lacuna no escopo da pesquisa.

### Contribuições para a área da Saúde e Políticas Públicas

O estudo oferece uma visão abrangente da parentalidade paterna em diferentes estágios da vida e contextos profissionais. Também evidencia a falta de preparação formal para o exercício da parentalidade paterna. No contexto das políticas públicas, é possível destacar que os participantes reconhecem os desafios, a falta de preparo e tempo como obstáculos para a parentalidade paterna. Suas ações buscam promover o desenvolvimento integral dos filhos, incluindo estratégias cognitivas, socioemocionais e interação com a natureza. A falta de políticas específicas e a necessidade de orientação são pontos discutidos, evidenciando a importância da compreensão social e do papel dos profissionais de saúde na promoção da parentalidade paterna. Destaca-se a importância do envolvimento dos pais nas atividades que promovem o desenvolvimento integral das crianças, como estímulos cognitivos, jogos lúdicos, atividades artísticas e interações emocionais. Ações essas, fundamentais para o desenvolvimento cognitivo, emocional e social das crianças, contribuindo para o crescimento saudável e o fortalecimento dos vínculos familiares.

## CONSIDERAÇÕES FINAIS

Compreende-se que a experiência da parentalidade paterna é descrita como desafiadora e gratificante, sendo fundamental para o cuidado e proteção dos filhos. A falta de preparação e as restrições de tempo são reconhecidas como desafios significativos para o cumprimento dos papéis parentais dos homens. Dentre as ações que promovem o desenvolvimento infantil, os homens destacam a importância de atividades que potencializam o desenvolvimento cognitivo e socioemocional, como jogos, música, arte e interação com a natureza. O papel dos profissionais de saúde, especialmente os enfermeiros, é fundamental na orientação e envolvimento dos homens nos cuidados aos filhos, desde o pré-natal. Esse envolvimento pode resultar em pais mais preparados e sensibilizados para estimular positivamente o desenvolvimento infantil.

Este estudo ressalta a necessidade de políticas públicas que apoiem a parentalidade paterna, enfatizando a importância da participação nas consultas pré-natais e de puericultura, e a necessidade de proporcionar um ambiente emocionalmente seguro para a criança. Estudos futuros poderão aprofundar os efeitos a longo prazo da parentalidade paterna no desenvolvimento infantil, explorando áreas como o equilíbrio entre a carreira e as responsabilidades parentais.

## Data Availability

https://doi.org/10.48331/scielodata.OKGAXW
